# The importance of elders: Extending Hamilton’s force of selection to include intergenerational transfers

**DOI:** 10.1073/pnas.2200073119

**Published:** 2022-07-06

**Authors:** Raziel Davison, Michael Gurven

**Affiliations:** ^a^Department of Anthropology, University of California, Santa Barbara, CA 93106;; ^b^Broom Center for Demography, University of California, Santa Barbara, CA 93106

**Keywords:** human evolution, force of selection, intergenerational transfers, life history theory, postreproductive lifespan

## Abstract

In classical evolutionary models, the force of natural selection diminishes with age toward zero by last reproduction. However, intergenerational resource transfers and other late-life contributions in social species may select for postreproductive longevity. We present a formal framework for estimating indirect fitness contributions via production transfers in a skills-intensive foraging niche, reflecting kinship and cooperation among group members. Among contemporary human hunter-gatherers and horticulturalists, indirect fitness contributions from transfers exceed direct reproductive contributions from before menopause until ages when surpluses end, around the modal age of adult death (∼70 y). Under reasonable assumptions, these benefits are the equivalent to having up to several more offspring after age 50. Despite early independence, minimal production surplus, and a shorter lifespan, chimpanzees could theoretically make indirect contributions if they adopted reliable food-sharing practices. Our results for chimpanzees hypothetically adopting hunter-gatherer subsistence suggest that a skills-intensive foraging ecology with late independence and late peak production could select for human-like life histories via positive feedback between longevity and late-life transfers. In contrast, life history changes preceding subsistence shifts would not favor further life extension or subsistence shifts. Our results formalize the theory that longevity can be favored under socioecological conditions characterized by parental and alloparental care funded through transfers of mid- to late-life production surpluses. We also extend our analysis beyond food transfers to illustrate the potential for indirect fitness contributions from pedagogy, or information transfers. While we focus mostly on humans, our approach is adaptable to any context or species where transfers can affect fitness.

## Significance

Prominent explanations for postreproductive longevity emphasize the myriad ways in which older adults help descendants in social species. However, standard metrics expressing how natural selection acts with age show declines in tandem with reproduction, rendering postreproductive life vulnerable to harmful mutations. Here, we develop a framework for estimating three fitness metrics to characterize the “force of selection” in social species with pooled energy budgets. We show that intergenerational transfers of food and information in the complex, high-skill foraging niche typical of hunter-gatherers can select for longer lifespan via inclusive fitness benefits. Our findings support the theory that postreproductive life in some mammals coevolved with multigenerational cooperation in a complex foraging niche and help explain selection against late-acting deleterious alleles.

Human life history is distinguished from that of other primates by delayed sexual maturity, long juvenile dependency, high paternal investment and alloparental care, menopause, and extended postreproductive lifespan (1–3). Surplus food production in adulthood subsidizes preadults and arguably plays an important role in the evolution of human adult lifespan (2, 4). Such intergenerational transfers from older to younger generations may also shape mortality in other social species (5, 6). However, W. D. Hamilton’s (7) “force of selection” considers only direct reproductive contributions to fitness. When reproduction declines with age to zero, postreproductive life is rendered vulnerable to harmful mutations. Mutations occurring in this “selection shadow” are invisible to selection and can therefore lead to rapid increases in mortality with age. Such age-related declines in selection intensity underlie most evolutionary theories of senescence (8, 9). The prominence of human menopause combined with postreproductive longevity, unique among primates and unexplained by classical approaches (10), suggests that transfers and other social processes may alter selection in ways overlooked by classic evolutionary models.

To extend theories of selection to explain human longevity, we investigate feedbacks between life history and subsistence strategies, showing how indirect fitness contributions from transfers can select for longevity in humans and other social species with cooperative food sharing. Human life histories are “slower” than in other primates in terms of age at maturity and lifespan, but also “fast” due to having short interbirth intervals and high reproductive effort across a shorter portion of the life cycle (11). Survival tradeoffs with high fertility may keep infant mortality high (12), but alloparental care relieves some of the energetic burden of high fertility via in-kind transfers and assistance to mothers and children (1–3, 13). Also, long childhoods allow the cultivation of skills required for extractive foraging, but require transfers to cover the energetic costs of extended dependence. It has been proposed that investment in large brains and social learning are required to develop high-skilled foraging strategies that increase production returns and generate surpluses (2, 14–16). Because intergenerational transfers of adult surpluses can increase the fertility and survival of others (especially the young), these indirect fitness contributions could drive selection for survival well beyond ages of reproductive cessation (4, 17).

Here, we contribute to ongoing research investigating the critical role of skills-intensive foraging in modifying the force of selection that shapes the age profiles of survival and fertility that are fundamental to fitness. Somatic and reproductive senescence are largely coupled in nonhuman primates, whereas the extended postreproductive lifespan of humans is unique (18). While human menopause has been theorized to result from life history tradeoffs or intergenerational conflict (19), here we take observed patterns of reproduction as a given and model selection on extended postreproductive lifespan due to food and other transfers. Because transfers in food-limited populations increase fertility and survival of kin, they generate positive selection for longevity well beyond reproductive cessation. While others have recognized and formally modeled how transfers can select for longevity and for other features of human life history (2, 15, 17, 20), evolutionary theories of aging have been guided largely by Hamilton’s force of selection (7), often expressed as elasticities of fitness to age-specific mortality and fertility (21).

Our framework extends the force of selection to account for production transfers, even in the absence of reproduction at certain ages. Because transfers generate larger fitness gains when directed to biological kin and cooperating group members who reciprocate and/or adhere to social norms of sharing, our framework incorporates the kinship between donors and recipients of different ages and the reliability of cooperation. If kinship is low and cooperation minimal, even large productive surpluses may not generate fitness advantages to longer life. Indeed, simulations show that indiscriminate sharing in the absence of reciprocity can help decrease juvenile mortality, but does not select for postreproductive longevity (22).

If we consider a “focal group” as a kin-structured sharing group, then adults contribute to their own inclusive fitness both directly through reproduction and indirectly via transfers. If production transfers by postreproductive and nonreproducing individuals increase fitness by improving female fertility and offspring survival, then age profiles of skills-intensive production showing peaks in midlife to later life should select for survival at older ages and increase representation of skilled, postreproductive adults. In this paper, we provide “proof of concept” for a hypothesis that late-life production surpluses of skilled foragers and subsistence farmers contribute indirectly to fitness through the nutritional effects of transfers.

To put human life history in an evolutionary context, we compare the potential for direct vs. indirect fitness contributions among wild chimpanzees, human hunter-gatherers, and horticulturalists. Chimpanzees are the closest surrogate for the last common chimpanzee–human ancestor 5 to 7 million y ago (23). Chimpanzees are self-sufficient foragers a few years postweaning and generally do not produce large food surpluses or transfers beyond those made to offspring, allies, and mates (2, 24). Chimpanzee feeding ecology is therefore not expected to generate selection pressure for late-adult survival. In contrast, hunter-gatherers rely heavily on others for up to two decades, but then produce surpluses through adulthood and into late life (2, 25). Food sharing is widespread among hunter-gatherers (24), within and between generations, with food directed to family, neighbors, friends, and others in ways that reduce food shortfalls at different timescales (4). Food is not the only type of transfer that impacts fitness: Allocare, conflict resolution, and different forms of information transfer are commonly observed in hunter-gatherers (1, 26). These nonfood transfers made by older adults confer additional fitness benefits, but those benefits can be difficult to measure, especially in observational studies. Despite this methodological limitation, identifying adult ages where net fitness benefits diminish rapidly would help distinguish between different proposals for the evolution of postreproductive lifespan in humans but not in other apes. For example, proposed late-age fitness benefits may only accrue up until a parent’s last child reaches sexual maturity (i.e., “mother hypothesis”) (27) or may include survival advantages from helping young grandchildren (i.e., “grandmother hypothesis”) (11). Also, because some skill-intensive production strategies, including many horticultural tasks, are less dependent on strength and agility, horticulturalists may provide surpluses at later ages than hunter-gatherers (28, 29).

Our study builds upon previous work examining feedbacks between life history, food production, and transfers (15, 17, 22, 30). While previous studies generate findings consistent with an altered force of selection on postreproductive survival, they do not permit age-based comparisons of contributions to inclusive fitness or directly model indirect fitness contributions. Our approach models food production, transfers, and vital rate responses explicitly. Given age profiles of production (*P_x_*), caloric demand (*D_x_*), fertility (*m_x_*), and survival (*p_x_*) and flexible assumptions about the nutritional dependence of vital rates, the relatedness, and the degree of reliable cooperation among members within the focal group, our model estimates the potential for inclusive fitness benefits through food transfers between individuals of different ages.

We characterize three fitness measures: indirect fitness contributions via production transfers, the fitness elasticity to production, and expected residual fitness contributions from future production transfers (“productive value”). Indirect fitness contributions (Δ*λ_Px_*) are the marginal inclusive fitness benefits provided by production transfers from individuals of different ages that would increase the population growth rate *λ* of a hypothetical focal sharing group (*λ* = 1 at stationarity). These are comparable to the direct reproductive contributions to *λ* made by fertility and survival at different ages. Fitness elasticities to production (*e_Px_*) predict the percentage of change in *λ* due to a 1% change in production *P_x_* and indicate the force of selection for survival on the basis of indirect fitness contributions made via production transfers. Because these elasticities do not sum to unity, these are not directly comparable to survival and fertility elasticities (21), but we compare relative age profiles to identify ages where selection is expected to be stronger than at other ages. Finally, productive value (PV) estimates the expected fitness value of future lifetime production transfers assuming survival to a given age and is comparable to Fisher’s reproductive value (31), which sums the reproductive contributions remaining over the life course after taking future mortality into account. Our flexible but conservative approach examines the fitness value of transfers across a wide range of vital rate responses to food availability across the observed range of mean intragroup relatedness, and with varying degrees of cooperation. Following ref. 32, we also look beyond food production to consider the role of information transfers on fitness via the effect of teaching on lifetime food production.

To demonstrate the utility of our approach, we evaluate the force of selection in wild chimpanzees, hunter-gatherers, and horticulturalists. We then employ these results to make inferences about the role of foraging ecology in shaping human life history evolution.

## Results

### Overview.

We estimate indirect contributions made via food sharing, demonstrate the force of selection acting on survival through production transfers, and characterize the residual fitness benefits of food transfers. Fitness contributions depend on the functional relationship between nutritional intake and vital rates, as well as kinship and cooperation among the sharing group. Skill-intensive subsistence strategies have a larger potential for indirect fitness contributions due to longer child dependence and larger late-life surpluses. Chimpanzee production is less skill intensive than human production and shows lower potential for indirect fitness contributions. However, a counterfactual chimpanzee life history combined with a hunter-gatherer production profile allows sizeable indirect contributions if chimpanzees reliably pooled food and shared widely. In contrast, the potential for indirect fitness contributions of horticulturalists is lower than among hunter-gatherers despite larger late-life surpluses because there is less room for nutritional improvement in their already higher vital rates. In the next section, we lay out our approach for linking transfers to changes in vital rates, to estimate our three fitness metrics.

### Nutritional Response of Vital Rates.

**Fig. 1.**
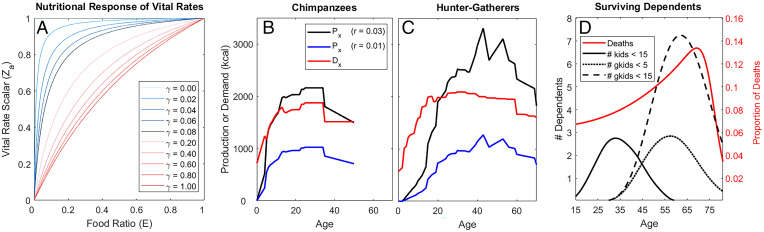
Nutritional response of vital rates, production scaling, and surviving dependents. (*A*) Vital rate scalar, *Z* = *E*(*γ* + 1)/(*E* + *γ*), increases mortality and decreases fertility when the food ratio (*E*) is below replete (i.e., *E* < 1) (main text). Lines show different nutritional responses under different curvature parameters (*γ*). (*B*) For a given survival response (here *γ* = 0.3), chimpanzee production (*P_x_*) is scaled to meet population demand (*D_x_*) at “replete” nutrition yielding the maximum nutritional-dependent population growth rate of 3% (*E* = 1, *C_T_* = *D_T_*, *r* = 0.03; black lines) or scaled for baseline” initial conditions (*E* < 1, *C_T_* < *D_T_*, *r* = 0.01; blue lines). (*C*) Hunter-gatherer production is similarly scaled (*γ* = 0.3). (*D*) Red line shows the distribution of adult lifespans for average hunter-gatherer demographic profile. Solid black line shows average number of dependent children (<15 y) to a focal mother; other lines reflect number of grandchildren, either <5 y old (dashed line) or <15 y old (dotted line).

We start with a focal sharing group that pools production (*P_x_*) from individuals at all ages (*x*) and redistributes it across the age schedule according to caloric need (*D_x_*). Following refs. 33 and 34, we characterize the nutritional state of a focal group through its food ratio (*E*), defined as the ratio of total caloric production at the level of the sharing group (*P_T_* = **Σ_x_** *P_x_ l_x_*) to total caloric demand (*D_T_* = **Σ_x_** *D_x_ l_x_*) across all ages *x* (*E* = *P_T_*/*D_T_*) variables defined in (see *SI Appendix*, Table S1 for complete variable definitions). In a need-based sharing regime, all individuals are allocated the same proportion of their demand regardless of age, so *E* at each age is the same. Vital rates and the population growth rate (*λ*) are maximized at *E* = 1, where demand is fully met by production (*P_T_* = *D_T_*), whereas lower consumption and unmet caloric demand reduce fertility and increase mortality by a scalar multiplier *Z*, with a less concave (more linear) response when the curvature parameter *γ* is large (Fig. 1*A*) (*Materials and Methods*). The minimum food ratio (*E_min_*) reflects population collapse (*r* < 0). Age-specific survival and fertility, *p_x_*(*E*,*γ*) and *m_x_*(*E*,*γ*), are functions of *E* and *γ* and are used to calculate all demographic variables: survivorship to age *x* (*l_x_*), net reproductive rate (*R*_0_), asymptotic growth rate (*λ*), intrinsic growth rate (*r* = log *λ*), life expectancy (*e*_0_), and total fertility rate (TFR) (*SI Appendix*, Figs. S1 and S2).

Our approach first considers a replete population (*E* = 1) at its maximal growth rate (*r* = 3% in chimpanzees and hunter-gatherers, *r* = 4% in horticulturalists) and then reduces production proportionally at each age *x* until the intrinsic growth rate (*r*) is diminished to 1% (at *E* = *E*_0_), the average growth rate for contemporary hunter-gatherers (35) (*SI Appendix*, Fig. S3). Starting with a baseline life history and subsistence profile driving 1% annual growth, we estimate selection on production transfers and potential indirect fitness contributions using serial sensitivities assuming different nutritional response curvature parameters *γ* (Fig. 1*A* and *Materials and Methods*).

### Inclusive Fitness and Cooperation.

To examine how intragroup relatedness influences the inclusive fitness effects of transfers, we discount indirect fitness contributions by Hamilton’s relatedness coefficient (*r_x_*), reflecting the mean relatedness of a donor age *x* to the donor’s focal sharing group (a smaller, more closely related subset of the donor’s residential group). Among hunter-gatherers and horticulturalists, the average relatedness among all residential group members is about 0.08 to 0.11 (36). We examine the effects of average relatedness within focal sharing groups, which is higher. For example, average relatedness among members of sharing clusters among Mbendjele BaYaka and Agta foragers is close to 0.2 (37). Here, we let relatedness within our focal sharing groups range from very low (*r_x_* = 0.001) to very high (*r_x_* = 0.5, where all group members are the equivalent of a full sibling, parent, or offspring) (*SI Appendix*, Fig. S4). Mean relatedness is generally higher in smaller kin groups, when dispersal is low, and when reproductive skew is high. Age profiles of relatedness among small-scale human societies also vary by group size and marriage residence patterns (38, 39). In small residential groups of ∼25 people, average relatedness can be as high as 0.20 (*SI Appendix*, *Additional Methodological Details and Results*). When relatedness to other group members increases with age, indirect contributions are larger overall, but smaller in early life (*SI Appendix*, Fig. S5*A*). Due to higher fitness sensitivities in early life, preferential sharing to children over adults would increase the benefits of transfers beyond that shown here, especially if better nutrition reduces age at menarche. The reinforcing effect of intragroup cooperation is included as a discount reflecting the average probability that an individual contributes the same transfers at older ages that the individual received at younger ages (e.g., *k* = 0.5 means that individuals fully cooperate with sharing norms 50% of the time).

### Humans vs. Chimpanzees.

Chimpanzee production has relatively low skill requirements, with self-sufficiency achieved by age 5 y, well before onset of reproduction (2). Human food production increases slowly and peaks much later, with greater surpluses characteristic of a high-skill niche (Fig. 1*B* vs. Fig. 1C). Stylized models (40) comparing high- vs. low-skill hunter-gatherer foraging niches yield qualitatively similar results to our species-level comparison, with hunter-gatherers exhibiting skills-intensive foraging and chimpanzees relying less on skill (*SI Appendix*, *Additional Methodological Details and Results* and Fig. S6 A and *B*). Horticulturalists produce even greater adult surpluses than hunter-gatherers, with slower declines by age. Among chimpanzees, 21% of lifetime production remains after age 30 y (7% after age 40 y), but among hunter-gatherers 66% remains at age 30 y (45% at age 40 y) and among horticulturalists 64% remains at age 30 y (40% at age 40 y) (*SI Appendix*, Fig. S7 and Table S2). By age 50 y, only 2% of chimpanzee production remains, compared to 24% among hunter-gatherers and 21% among horticulturalists. Similar to reports across both high- and low-income countries (41), we find that the net flow of food transfers in our sample of hunter-gatherers and horticulturalists is downward, with the average age of production exceeding that of consumption by about a decade in humans (range: 5.6 to 13.6 y), but by only 2.6 y in chimpanzees (*SI Appendix*, Fig. S8). Also, the average age of human forager production exceeds the average age of reproduction by 7 to 15 y across our sample, whereas the average age of chimpanzee reproduction exceeds average ages of both production and consumption. Therefore, hypothetical chimpanzee transfers would mainly support early fertility, whereas hunter-gatherer transfers fund prime adult-age mothers and horticulturalist transfers support slightly older mothers.

### Direct vs. Indirect Contributions to Population Fitness.

**Fig. 2.**
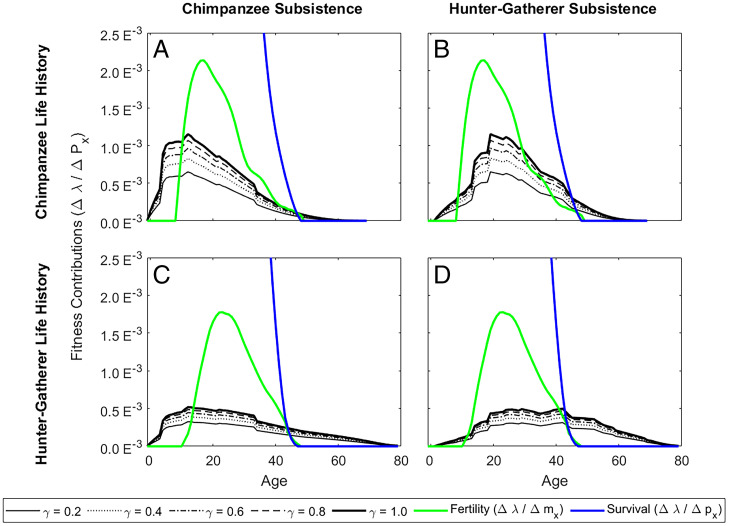
Age profiles of indirect vs. direct fitness contributions. Panels compare direct fitness contributions (Δ*λ_mx_*, green lines; Δ*λ_px_*, blue lines) vs. indirect fitness contributions via production transfers (Δ*λ_Px_,* black lines) under different nutritional responses (*γ*), with high relatedness (*r_x_* = 0.5) and perfect cooperation (*k* = 1). Panels show predictions for populations with (*A*) chimpanzee vital rates and subsistence, (*B*) chimpanzee vital rates and hunter-gatherer subsistence, (*C*) hunter-gatherer vital rates and chimpanzee subsistence, and (*D*) hunter-gatherer vital rates and production. Lines distinguish treatments with differing starvation responses (*ɣ*). For comparison, colored lines show direct contributions to population growth made via fertility (green) and survival (red).

Among chimpanzees, potential fitness contributions via production transfers are largest in early life (Fig. 2*A*), with high mortality allowing substantial fitness gains through improved nutrition. Thus, if chimpanzees pooled food on the basis of caloric need, rather than restricted feeding of juvenile dependents and select others (24), they could provide sizeable indirect fitness contributions despite their low-skill, low-surplus subsistence. However, even with widespread sharing (*k* ∼ 1) and a lack of overt menopause (10), the potential for late-life fitness contributions is low among chimpanzees due to both reproductive and actuarial senescence. With little social cooperation (low *k*), chimpanzee donors would benefit even less from pooling resources.

Because of higher human survivorship and greater adult production surpluses, both direct and indirect fitness contributions are larger among humans at older ages compared to chimpanzees (Fig. 2*D*), with similar age patterns observed across small-scale societies (*SI Appendix*, Fig. S9 *A–E*). By age 30 y, 66% of hunter-gatherers’ cumulative indirect fitness contributions remain (45% at age 40 y, 24% at age 50 y), whereas chimpanzees have 20% remaining at age 30 y, 7% at age 40 y, and only 2% at age 50 y (*SI Appendix*, Fig. S7 and Table S2). Horticulturalists are similar to hunter-gatherers, with 64% remaining at age 30 y, 40% at age 40 y, and 21% at age 50 y (*SI Appendix*, Fig. S7 and Table S2). Although horticulturalists have higher production surpluses than hunter-gatherers, indirect contributions among hunter-gatherers are slightly larger due to higher baseline mortality and lower early-life fertility (*SI Appendix*, Fig. S10). When transfers have a higher impact (larger *γ*), indirect contributions exceed fertility contributions at earlier ages (Fig. 2 and *SI Appendix*, Fig. S10). Overall, these metrics contrast strongly with direct fertility contributions over the life cycle, of which hunter-gatherers have only 37% and 8% remaining at ages 30 and 40 y, respectively; forager-horticulturalists have 30 and 5% remaining, but chimpanzees have only 20 and 4% left (*SI Appendix*, Fig. S7 and Table S2).

### Counterfactual Subsistence and Life History Profiles.

If delayed peak production, large midlife surpluses, and intergenerational transfers coevolved with longevity (2, 4, 17), then human-like lifespans should not be favored under a chimpanzee feeding niche. To gain insight into the coevolutionary sequence leading to human longevity, we examine two hypothetical cases. If chimpanzees retained their subsistence ecology but had the fertility and survival of hunter-gatherers, there would be higher potential for late-life production contributions due to higher survivorship (Fig. 2*C*). However, despite early foraging independence and hunter-gatherer longevity, reproductive maturity would be delayed relative to chimpanzees and chimpanzee production does not provide large adult surpluses to fund indirect contributions. On the other hand, if chimpanzees hypothetically adopted hunter-gatherer subsistence but retained their fertility and mortality profiles, the potential for indirect fitness contributions would be high and indirect contributions would outweigh fertility contributions at earlier ages (Fig. 2*B*), driving selection for longevity. This combination has the highest potential for indirect contributions because large adult surpluses are required to support juveniles up to foraging independence and because earlier fertility and high mortality of chimpanzees both allow strong payoffs to production transfers. Although this hypothetical example requires chimpanzees to be efficient at hunting and gathering and to possess the cognitive capacity, social institutions, and individual motivation to facilitate pooled food sharing, this combination illustrates the potential for large indirect contributions, even when mortality is high and there is no menopause strictly limiting late-life reproduction. However, given chimpanzees’ lower foraging efficiency (217 kcal/h vs. 729 kcal/h for hunter-gatherers and 2,162 kcal/h for horticulturalists) (42), they would have to spend considerably more time foraging than hunter-gatherers to provide sufficient production transfers without a radical shift in foraging strategies (*SI Appendix*, Fig. S11).

**Fig. 3.**
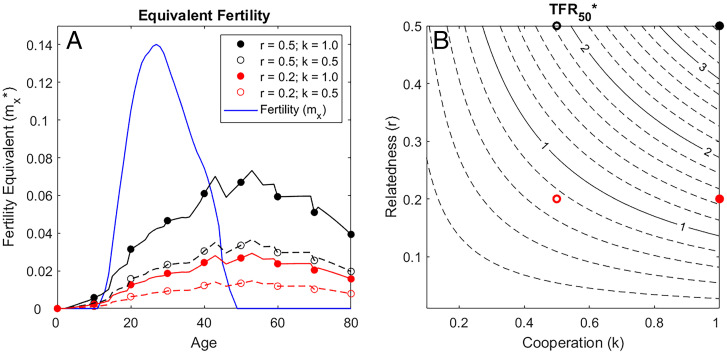
Indirect fitness contributions expressed as “equivalent fertility.” Assuming different levels of mean relatedness (*r_x_*) and cooperation (*k*) for the average hunter-gatherer life history and subsistence profile, (*A*) Equivalent fertility (m_x__*_) predicts the number of female offspring that would need to be produced at each age, given fertility sensitivities at those ages and a moderate starvation response (*γ* = 0.3), to yield direct fitness contributions equivalent to those predicted from production at those ages. Lines distinguish combinations where relatedness is high (*r* = 0.5) vs. low (*r* = 0.2) and cooperation is moderate (*k* = 0.5) or high (*k* = 1.0); blue line shows age-specific fertility (*m_x_*). (*B*) TFR_50_ represents the expected number of future offspring (of both sexes) that would be born to a mother living from age 50 y to age 80 y under the equivalent fertility schedule in *A*. Contours predict TFR_50_ under different combinations of cooperation (*k*) and mean relatedness to the group (*r*); markers indicate TFR_50_ for the examples in *A*.

To ease interpretation of effect sizes, we present indirect fitness contributions as fertility equivalents (m_x__*_) by calculating the additional fertility at each age that would yield the same fitness contribution, given sensitivity to fertility at that age. If cooperation is perfect (*k* = 1) and kinship is maximized (*r_x_* = 0.5, same as relatedness to genetic offspring), food transfers fund fitness contributions roughly equivalent to 0.04 to 0.06 daughters per year after age 40 y (Fig. 3*A*), resulting in about 3 to 5 additional children of both sexes from age 50 to 80 y, depending on the starvation response (Fig. 3*A* shows predictions for *γ* = 0.3). If mean relatedness is lower (*r_x_* = 0.2) and cooperation is moderately reliable (*k* = 0.5), production transfers after age 50 y would yield the equivalent of 0.01 daughters per year after age 50 y and 0.7 to 0.8 offspring of both sexes between ages 50 and 80 y (Fig. 3*B*).

### Fitness Elasticities to Production.

**Fig. 4.**
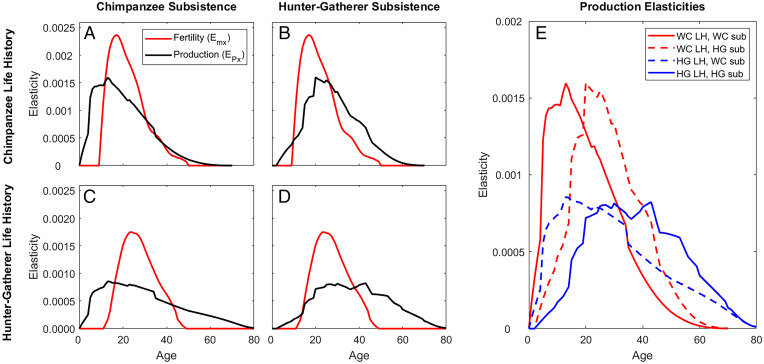
Fitness elasticities in humans and chimpanzees. For pairwise combinations of chimpanzee life history (*p_x_*, *m_x_*) and subsistence profiles (*P_x_*, *D_x_*), fitness elasticities to fertility (*e_mx_*) and production (*e_Px_*) are shown, each scaled to sum to unity for comparison of age profiles (**Σ_x_** *e_mx_* = **Σ_x_** *e_Px_*). Examples shown apply moderate nutritional response (*γ* = 0.3), kinship (*r_x_* = 0.2), and cooperation (*k* = 0.5). (*A* and *B*) show results for chimpanzee life history and (*C* and *D*) show results for hunter-gatherer life history; *A* and *C* show results for chimpanzee subsistence and *B* and *D* show results for hunter-gatherer subsistence. (*E*) Elasticities to production are compared across combinations of hunter-gatherer vs. chimpanzee life histories (labeled LH) and subsistence profiles (labeled sub) (*γ* = 0.3 in example).

Fitness elasticities predict proportional changes in fitness due to proportional changes in reproduction or production, and elasticities to production (*e_Px_*) tell us about the relative potential for indirect contributions at different ages. Because production elasticities are not independent of fertility and survival elasticities (which sum to 100% of all potential direct fitness contributions), we cannot compare them directly. By scaling them to sum to the total elasticity to fertility, however, they can be compared across populations to reveal the relative potential for indirect contributions at different ages (Fig. 4*E*). Elasticity to early production is relatively lower among humans than among chimpanzees because of extended child dependency, but higher at late ages at which humans have larger production surpluses and higher survivorship (Fig. 4). Production elasticities peak later in humans than in chimpanzees (Fig. 4) for two reasons: 1) High chimpanzee mortality limits the potential for late-life contributions, and 2) caloric demand is lower among chimpanzees relative to humans, so there is relatively less room for improvement through production transfers.

Even if chimpanzees could organize pooled sharing, we find that selection for production in chimpanzees falls off at the same relative rate as selection for fertility (Fig. 4*A*), whereas production elasticities remain high into old age for hunter-gatherers (Fig. 4*D*). However, if the chimpanzee subsistence profile was like that of hunter-gatherers, late-age production elasticities would be higher, extending beyond the period of fertility decline (Fig. 4*B*), and would drive selection for improved adult survival, including postreproductive survival. In contrast, human hunter-gatherers with a chimpanzee-like foraging niche would have less production incentive in midlife because fertility is still relatively high, but selection on production would also extend farther because of higher survivorship at older ages (Fig. 4*C*). These results are robust to a wide range of nutritional responses to transfers (*SI Appendix*, Fig. S9 F*–*J).

### Prospective Fitness Value.

**Fig. 5.**
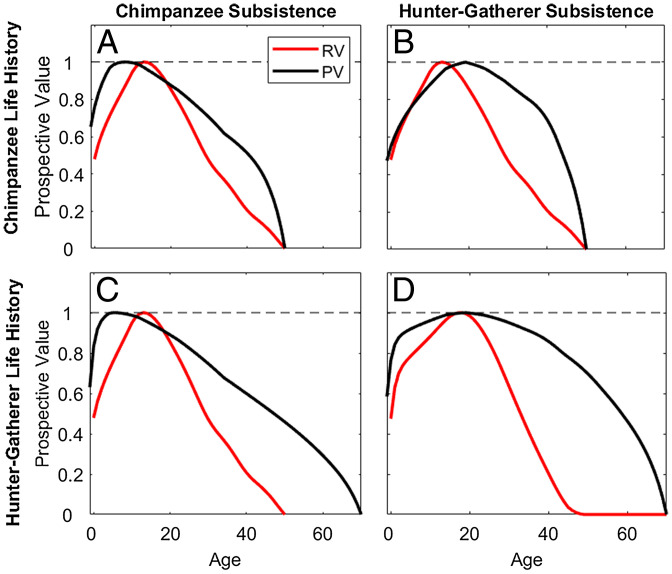
Reproductive value vs. productive value. For pairwise combinations of chimpanzee life history (*A* and *B*) vs. hunter-gatherer life history (*C* and *D*) and chimpanzee subsistence (*A* and *C*) and hunter-gatherer subsistence (*B* and *D*), lines show prospective fitness value (reproductive value vs. productive value under different survival responses). Examples shown apply moderate kinship (*r_x_* = 0.2) and cooperation (*k* = 0.5). Since PV is a scaled metric, altering nutritional responses (e.g., *γ* = 0.2 to 1.0) does not affect results.

We compare the expected future fitness value of reproduction (i.e., Fisher’s reproductive value [RV]) to the residual fitness value of caloric production (PV) (Fig. 5 and *SI Appendix*, Fig. S9 *K–O*). Among chimpanzees, PV is high at young ages due to early foraging independence, and RV decreases faster than PV due to declining fertility (Fig. 5*A*). PV is higher than RV among humans over much of adulthood and declines much more slowly with age due to sustained surpluses well beyond reproductive cessation at age 50 y (Fig. 5*D*). Chimpanzees hypothetically adopting hunter-gatherer subsistence would have higher PV that exceeds RV by early adulthood, but would still be limited by high mortality (Fig. 5*B*). Among hunter-gatherers with chimpanzee-like subsistence, PV declines more slowly with age due to lower mortality, but is lower at prime ages due to smaller surpluses (Fig. 5*C*). As with indirect fitness contributions, PV of older individuals is higher under scenarios when mean relatedness to other focal group members increases with age (*SI Appendix*, Fig. S5*B*).

### Pedagogy Contributions.

**Fig. 6.**
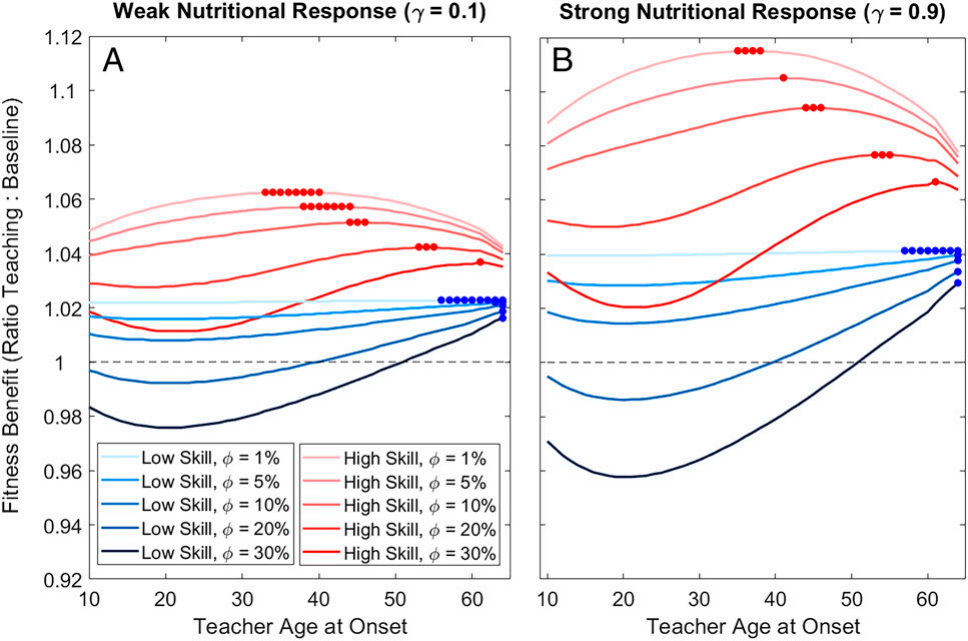
Fitness contributions of pedagogy. Increases in population fitness are shown for pedagogical investments under high-skill (red) vs. low-skill (blue) subsistence, assuming pupils learn skill twice as fast (*θ* = 100%) during 10 y of instruction beginning at age 10 y. Assuming either (*A*) weak nutritional response of vital rates (*γ* = 0.1) or (*B*) strong nutritional response (*γ* = 0.9) and low production costs to teachers (*φ* = 1, 5, 10, 20, or 30%; line shades). Horizontal dashed line indicates the break-even thresholds (zero fitness gain from pedagogy); solid circles indicate optimal ages of instruction.

Although our empirical approach focuses on food production and food transfers, it is generalizable to other types of transfers impacting vital rates. Here, we also consider information transfers, which include teaching, pedagogy, and instruction. Although there is debate about the degree of direct instruction that occurs across small-scale societies, current evidence supports a wide range of behaviors (e.g., feedback, demonstration, opportunity scaffolding, pointing, etc.) that serve to improve the skills of others (43). In a prior model (32), we showed how pedagogy can improve food production skills, especially in high-skill feeding niches. Here, we estimate the fitness contributions of pedagogy using an illustrative example, applied to our composite hunter-gatherer life history. If pupils receive 10 y of direct instruction beginning at age 10 y and pupils under instruction learn skills twice as fast, we estimate the potential for indirect fitness benefits through pedagogy, assuming different nutritional responses (γ), costs to teachers (*φ*), and teacher ages (*b*). With a weak nutritional response (*γ* = 0.1) and low costs of teaching (*φ* = 0.01), perfect cooperation (*k* = 1), and close relatedness (*r_x_* = 0.5), optimal pedagogy could increase the focal group fitness from 1 to 1.06% annual growth (Fig. 6*A*); with a strong nutritional response to production transfers (*γ* = 0.9), focal group fitness increases to 1.12% annual growth (Fig. 6*B*). As expected, greater fitness value to pedagogy occurs under a skills-intensive production niche, where teaching costs are low and where food transfers have stronger impacts on vital rates (high *γ*). As expected, optimal teacher ages are older when teaching costs are high.

## Discussion

Beginning with Medawar and Haldane, and later formalized by Hamilton, classical explanations for the evolution of senescence have emphasized age declines in the force of selection against deleterious alleles that increase late-age mortality or reduce late-age fecundity (44). Although specification of this force of selection varies (7, 45), it usually predicts a “wall of death” near the age of reproductive cessation, as selection intensity diminishes to zero, and in some cases, nonlinear dynamics drive rapid senescence even before reproduction ceases (46, 47). In the classical framework, synchronized somatic and reproductive senescence is compatible with the accumulation of late-acting deleterious alleles (“mutation accumulation”) or of alleles that are harmful late in life but increase fitness early in life (“antagonistic pleiotropy”) (48). However, these theories relying on the classical framework cannot easily explain postreproductive lifespan or late-age mortality plateaus. Nor can they explain why adult mortality increases exponentially only after age ∼30 y in humans despite decreasing reproductive value after maturity. Furthermore, Hamilton’s indicators of selection look remarkably similar even with large differences in human mortality and fertility (49, 50), limiting their utility in predicting intraspecific variation (51).

Our major modification of the force of selection addresses productive transfers affecting inclusive fitness through provisioning and protection of juveniles, allocare, marriage brokering, or any activity that affects kin fertility or survivorship (52). Even if mean relatedness is low, strong (enforced) norms of redistribution will ensure individual fitness benefits by reducing risks of defection. By borrowing and lending across the life course, transfers generate “pooled energy budgets” permitting evolution of a number of human traits, including early weaning, rapid offspring growth, and closer birth spacing as a result of multigenerational cooperation (53). Here, we follow recent efforts to consider how transfers shape the evolution of human lifespan (15, 17, 20, 22, 54, 55). Employing empirically derived food production profiles and demographic rates, we modeled the fitness effects of food transfers made via nutritionally mediated changes in vital rates. We modified three classic fitness metrics to estimate the potential for indirect fitness via production transfers: fitness contributions, fitness elasticities, and residual fitness value. All these fitness measures show that as reproduction declines but production surpluses continue, individuals’ inclusive fitness value to their sharing unit relies more on transfers that extend well beyond reproductive cessation. Of course, the relative importance of food transfers depends on the response of survival and fertility to nutrition, population age structure, and group characteristics (e.g., kinship and cooperativeness), which are flexible in our model.

Although the relationship between nutrition and vital rates likely depends on environmental conditions (e.g., prevalence of accidents or violence as additional sources of mortality), our qualitative findings vary minimally across a wide range of nutritional responses (*γ*) and across different populations of hunter-gatherers and horticulturalists (*SI Appendix*, Fig. S9). Young and middle-age adults ages 20 to 40 y show the largest indirect fitness contributions and elasticities to production, but older adults also make substantial fitness contributions despite mortality attrition. In our model, hunter-gatherers over age 50 y contribute almost a quarter of the total indirect fitness contribution despite accounting for only 11% of the population (*SI Appendix*, Fig. S7). With no age pattern in mean relatedness or cooperation (flat *r_x_* and *k* profiles), changes in mean relatedness and cooperation affect the magnitude but not the proportion of total contributions made after age 50 y. However, *r_x_* increasing with age would lead to higher, and decreasing *r_x_* would lead to lower, relative contributions made after age 50 y (*SI Appendix*, Fig. S5). The contributions made after the mean age of last reproduction (ages 35 to 42 y among small-scale human societies) represent an even larger proportion of the total (*SI Appendix*, Fig. S7 and Table S2). Across the life course, transfers allow nonreproducing and postreproductive adults to contribute fitness to the population.

Production data on elders over age 70 y is sparse, but both systematic and anecdotal evidence suggests steep declines in the 60s for hunter-gatherers and in the 70s for horticulturalists (25, 40), very near the modal age of death among subsistence populations, between ages 65 and 75 y (56) (Fig. 1*D*). Beyond these ages, indirect fitness contributions from transfers are small (Fig. 3) due to mortality attrition limiting the representation of older ages as well as rapidly declining surpluses at late ages. The number of dependent kin also drops steeply around this age (57). Among hunter-gatherers and horticulturalists, the number of dependent children (<15 y) peaks around the mid-30s and drops to zero by age 60 y, while the number of dependent grandchildren peaks by the mid-60s and declines steeply thereafter (56), with the pool of close descendants becoming small by the modal ages of death in the eighth decade of life (Fig. 1*D*). Even in many modern nation states, the degree of upward transfers from younger to older increases after age 75 y (58).

The large potential for indirect fitness contributions through production transfers supports evolutionary models showing that transfers could drive life history evolution from that of an ancestor inhabiting a low-skill chimpanzee-like production niche to one typical of contemporary hunter-gatherers (2, 17, 54). Chimpanzees’ early self-sufficiency and limited surplus constrain indirect fitness contributions even if they adopted kin-directed food sharing, but subsistence shifts could select for greater adult lifespan. Accordingly, chimpanzee fitness contributions, elasticities, and prospective fitness values in late adulthood are small (Figs. 2*A*, 4*A*, and 5*A*), even with lower adult mortality typical of hunter-gatherers (Fig. 2*C*). However, shifting to a more skills-intensive foraging niche would have increased the potential for large indirect fitness contributions (Figs. 2*B* and 4*B*). Delayed peak production and large late-life surpluses would fund intergenerational transfers, while increasing longevity would reinforce this process by preserving older skilled individuals.

Although causality is difficult to ascertain, especially given the ratchet-like coevolution proposed between subsistence strategies, adult survivorship, and sociality (2), our results suggest the shift to a more skills-intensive ecology preceded the evolution of long lifespan and delayed maturity. The fitness payoff to a chimpanzee-like ancestor adopting a subsistence strategy emphasizing high-return extraction or pursuit, combined with strategic kin-directed sharing (active or passive) and social mechanisms reinforcing cooperation, could select for increased longevity through mid- to late-life production transfers; extended provisioning while dependent juveniles learn foraging skills can also favor delayed maturity (2). Although chimpanzees share food with offspring, allies, potential mates, and occasional others through tolerated scrounging and some exchange, chimpanzees seem to lack the social disposition, psychological motivation, and possibly the cognitive capacity for more strategic food sharing and enforcing social norms (24, 59). Comparison with other cooperatively breeding primates suggests feedback between cognitive and social evolution and cooperative breeding (60). Without reliable food distribution or helping behavior, chimpanzees forfeit this potential for greater fitness benefits and risk pooling. Our findings suggest that the trajectory from chimpanzee-like to human-like subsistence niche was likely incremental, with increases in late-life production favoring kin-directed transfers through inclusive fitness benefits and from norm-based cooperation, in turn driving selection for longevity that increases the fitness value of late-life transfers.

A hunter-gatherer subsistence strategy with delayed foraging independence and mid- to late-life production surpluses may indeed require coevolution with lower adult mortality to reap the surplus gains achieved later in life (2, 55). Part of what makes hunter-gatherer livelihoods viable in the first place is buffering the risk of production failure through transfers—not only to children, juveniles, and adolescents (the “net consumers”), but also among adult producers with peak production but high child dependency (61, 62). For instance, net caloric deficits are common in over half of Ju/’hoansi, Ache, Hiwi, and Tsimane families with child dependents (4, 25, 62), so those without dependents are valuable contributors of food, allocare, and aid (3, 62). Kinship and relative need, determined by recipient age, productivity, family size, and health status, are salient predictors of greater resource flows (63), including upward transfers to older adults (29, 58). Human sociality also functions to aid in recovery from illness and to offset production losses experienced during disability (4, 64). This is important in foraging economies with high-variance returns because they rely on difficult-to-acquire bonanzas of meat and fish and extracted items like honey, larvae, and roots. For example, Hadza big-game hunters, Hiwi hunters, and Ache hunters are unsuccessful on 96, 65, and 40% of hunts, respectively (62).

In addition to resource pooling to buffer production failures, sharing also occurs within task groups dividing labor, especially by sex. Among hunter-gatherers, men more often target high-variance, high-energy foods like wild game, whereas women tend to focus on more predictable foods like shellfish and fruits (65). For simplicity, our empirical analysis considered only female food production curves. While Ju/’hoansi production profiles are similar among the sexes (25), late-age male surpluses are much larger for Ache, Hiwi, and Tsimane (2, 4). As ours is not a two-sex model (66), we do not incorporate sex differences in food production. Our use of only female age profiles of production and consumption therefore provides a conservative estimate relative to a mixed strategy with male production, which may be lower in late life but higher at peak ages (2, 4).

Even when kinship is relatively low, multigenerational sharing and cooperation are still common in small-scale human societies (24, 63). In larger groups, sharing tends to occur more among restricted networks of close kin and trustworthy partners, consistent with the notion of “focal group” used here. For humans with skills specialization and divisions of labor, production transfers extend beyond food, with other material and land transfers becoming especially important for reproductive success in farming populations (67). In addition, elders possess valuable knowledge and skills that can help increase group production even after reaching production deficits (32). Expertise in a wide range of fitness-relevant tasks (e.g., manufacturing, childcare, conflict mediation, healing, leadership) is frequently reported among middle-aged or older adults (68), and a previous study (32) predicted substantial increases in lifetime production with pedagogy, especially from older adults past prime childbearing. As a first step in considering other types of transfers, we modeled pedagogy as a type of information transfer. However, in our simple framework, the effects of pedagogy operate only through improving foraging skills and lifetime production, conservatively underestimating other avenues by which pedagogy could enhance survival and reproduction.

Our study has implications beyond humans, as skills-intensive subsistence regimes and sociality are also associated with more transfers and slower life history traits across species. Mammals with greater feeding-niche complexity reach adult-level skill competence closer to the age of first reproduction, subsidized in part by postweaning provisioning, whereas cooperative hunting species with greater resource sharing (e.g., gray wolves, bottlenose dolphins, spotted hyenas) peak in foraging competence after the age of first reproduction (69). Slow development in primates and provisioning in social carnivores both help to buffer low productivity during extended learning periods in species with complex feeding niches (70). Sociality is necessary for any reliance on transfers and itself may select for greater longevity by reducing mortality rates from predation and disease (71). Finally, the role of transfers, divisions of labor, and nonreproductive fitness contributions have been posited to help explain patterns of senescence in eusocial insects (6), where most individuals have zero direct fitness but workers make valuable contributions through transfers. That said, the fitness advantage of longevity exists largely for reproductive individuals and as far as we are aware, social naked mole rats (family *Bathyergidae*) may be the only eusocial species where workers sometimes live as long as queens (71) (but see ref. 72).

Postreproductive survival is documented in a number of mammals (73), but a “true” postreproductive life stage (i.e., not simply an artifact of senescence) is rare in mammals (10), limited to humans, short-finned pilot whales (*Globicephala macrorhynchus*), and resident killer whales (*Orcinus orca*) (74). Measuring the fitness value of postreproductive survival is difficult, but evidence to date is suggestive. Postreproductive orca females lead their offspring (and sometimes grandchildren or younger siblings) in collective foraging bouts, especially during periods of low salmon abundance (5). For these and other reasons, orca grandmothers generally increase grandoffspring survival (75).

Even without menopause, the force of selection via indirect contributions may be substantial in later adulthood where reproductive rates are low, particularly in long-lived social species. For example, long-lived Asian elephants (*Elephas maximus*) have highly dependent offspring but reproductive and somatic senescence are not decoupled (76). Nonetheless, Asian elephant grandmother coresidence shortens the daughter’s interbirth intervals and increases grandoffspring survivorship (77). While our fitness metrics reflect indirect fitness contributions even when reproduction is low or minimal, explanations invoking fitness costs of continued reproduction [e.g., from intergenerational conflict (74) or late-life pregnancy complications (19)] may be necessary to explain the evolution of menopause.

### Model Limitations.

Our model shows a proof of concept that intergenerational transfers can generate sufficient indirect fitness contributions to drive selection for longevity well beyond reproductive cessation, but under several simplifying assumptions. First, because we use matrix projection models that estimate mean effects from aggregated data, we do not examine the consequences of individual or group-level heterogeneity. Extensions of our analysis could explore the role of sexual division of labor (with distinct vital rates and production/consumption profiles of males and females) and skills specialization where individuals differ in their production returns for different subsistence activities (e.g., hunting, gathering, fishing, gardening). An alternative approach could use agent-based models to examine the optimal strategies of individuals in light of population age and kin structure. Cultural group selection models could demonstrate another way that prosocial, kin-directed, and needs-based transfer systems could proliferate in a metapopulation of groups with different social institutions. Second, our cooperation term *k* discounts benefits only when transfers to others in the focal group are not reciprocated or paid forward. We thus ignore internal group dynamics and other factors that affect intragroup cooperation. The ability to endogenize sharing rules by examining the consequences of defection in a modeling framework, rather than assuming the rules we do here, would be an important advance, but outside the scope of our study. Agent-based models and simulation could also explore other means of maintaining cooperation, such as prosocial reputation, punishment, and social information sharing.

## Conclusion

While food and even nonfood transfers in social species are not rare (78, 79), humans are unique in the breadth and volume of transfers across different domains and in the social mechanisms that limit defection and reinforce cooperation. Intergenerational transfers help explain the combination of slow and fast characteristics of human life history. The slow elements (delayed maturity and longevity) permit (and also require) extended learning of subsistence skills during a prolonged preadult life stage, whereas transfers also fund the fast elements of early weaning and overlapping child dependency. Transfers also facilitate rapid population growth during recovery from periodic catastrophes (35).

Why are intergenerational transfers not ubiquitous if they are so beneficial? First, transfers are often costly to donors, but may be less so when donors enjoy surpluses. The ability and motivation to generate surplus in a complex subsistence niche may be a prerequisite for transfers. Second, donors can recoup losses by targeting kin so benefits increase inclusive fitness and via complex cooperation (e.g., reciprocity, group augmentation, norm enforcement). Social systems fostering multilevel complex cooperation help make intergenerational transfers more profitable, and in our model, transfers allow adults to “pay forward” the assistance they received before independence by transferring to their children in the same fashion their parents transferred to them. Although fitness benefits are higher when sharing with close kin, and even more so if average relatedness increases with age, we also find substantial benefits in loosely related groups when cooperation is reliable and effective. This highlights the important role of social factors as necessary prerequisites for the evolution of longevity, at least in some species, like humans. With greater dependency and higher risk of production failures, complex subsistence niches provide opportunities for transfers to have large fitness impacts among the “have nots,” with inclusive fitness and social benefits accruing to donors. Indeed, kin help, especially by postreproductive grandparents, improves grandoffspring survivorship and/or daughter’s fertility in many cultural settings (80, 81). Even at the contemporary national level across 34 countries, public and private intergenerational transfers are associated with lower mortality and greater longevity (82).

The declining force of selection is often invoked as a reason why many diseases show a rapid increase in incidence across late life. The potential for indirect fitness impacts throughout adulthood, as well as nonzero selection at postreproductive ages, may provide additional insight into the age profiles of disease expression. Selection for postreproductive survival should alter expectations of deleterious allele frequencies based on mutation accumulation or antagonistic pleiotropy and of disease age-at-onset profiles (20, 83). Recent attempts at uncovering signals of viability selection for deleterious late-acting alleles in humans found very few common variants (only 2 of a possible 8 million), suggesting that nonzero purifying selection has weeded out many late-acting harmful alleles (84); our model suggests a plausible mechanism. It is our hope that exploration and application of nonclassical selection pathways, like those we investigate here, should help further illuminate the evolution of lifespan and aging-related diseases in social species.

## Materials and Methods

We construct a nutrition-dependent life history model to estimate indirect contributions of production and production elasticities at each age. We also derive age profiles of productive value, directly analogous to Fisher’s reproductive value (11), but reflecting indirect fitness contributions via production transfers rather than reproduction.

### Nutritional Dependence of Vital Rates.

Vital rate responses to nutrition are likely concave, with minor food deprivation having small costs that increase sharply with greater deficits (85, 86), an assumption supported indirectly through mortality responses to temporal changes in food prices (87). Given uncertain relationships, we vary the curvature of vital rate responses to nutrition from strong, nearly linear responses to weak responses where severe deprivation is required for large effects. This ensures that our qualitative findings are robust to a wide range of nutritional responses. Also, because we assume food-limited populations, we do not consider harmful effects of “hypernutrition” or attempt to identify the actual threshold of replete nutrition, instead assuming that “replete” nutrition would drive vital rates near the maximum observed (3% for hunter-gatherers and chimpanzees, 4% for horticulturalists) (50).

Nutritional status is modeled using the food ratio *E*, which reflects the ratio of total caloric production (*P_T_* = **Σ_x_** *P_x_ l_x_*) to total demand in the sharing group (*D_T_* = **Σ_x_** *D_x_ l_x_*) (33, 34); higher values of *E* describe a more nourished population. We conservatively assume equal food sharing according to caloric need without respect to age, so the food ratio for every individual is equal to total population production divided by total demand (*E* = *P_T_*/*D_T_*). Although the reaction norm of vital rate responses to nutrition is poorly resolved, it is reasonable to assume that the response is a convex saturating function with small effects of minor deprivation and increasingly large effects of severe malnutrition (85, 88). Therefore, we model the curvature of this norm of reaction using the parameter *γ*, where the response of the vital rate scalar *Z* to the food ratio *E* is more linear under higher values of *γ* (Fig. 1*A*):

[1] $$Z\;=\;\;\frac{E\left(\gamma +1\right)}{E+\gamma }.$$

We model the nutritional response of vital rates by applying the scalar *Z* to baseline fertility *m*_x_^(0)^ [i.e., *m*_x_(*E*,*γ*) = *Z*(*E*,*γ*) *m*_x_^(0)^] and applying 1/*Z* to baseline mortality *q*_x_^(0)^ [i.e., *q*_x_(*E*,*γ*) = *q*_x_^(0)^/*Z*(*E*,*γ*)]. We assume that vital rates and population growth rates reach their maximum (3% annually for hunter-gatherers and chimpanzees, 4% for horticulturalists) when nutritionally dependent vital rates are at their maximum (*E* = 1, *Z* = 1) (Fig. 1*B*). This gives us a benchmark to calculate the age profile of production that would meet total population demand. Under these nutritionally replete conditions, vital rates are maximized and additional caloric intake will not benefit survival or reproduction. We calibrate the initial conditions for our analysis with production yielding a baseline food ratio (*E*_0_) corresponding to 1% annual population growth, the contemporary hunter-gatherer average (35) (Fig. 1*C*). At this baseline, marginal nutritional status responds to changes in food availability as specified above (Fig. 1*A*). Assuming a given nutritional response (*γ*), the food ratio corresponding to stationary growth (*λ* = 1, *r* = 0) is *E_min_*(*γ*), below which the population is assumed to crash.

### Inclusive Fitness and Cooperation.

We discount indirect fitness contributions by Hamilton’s relatedness coefficient (*r_x_*), reflecting the mean relatedness of an individual age *x* to the focal sharing group (averaged across its stable age structure); *r_x_* ranges from very low (*r_x_* = 0.001) to very high (*r_x_* = 0.5). For ease of comparison with fertility contributions, all contributions are scaled relative to the relatedness of direct offspring (*r_x_* = 0.5) (*SI Appendix*, Fig. S4A). The cooperation coefficient *k* reflects the probability that a group member shares production according to the needs-based sharing rule (0 ≤ *k* ≤ 1). Complete defection would be *k* = 0, whereas complete compliance with sharing norms is *k* = 1. We apply *r_x_* and *k* to age contributions of indirect fitness across these ranges to show how kinship and cooperation affect our predictions. Age patterns of relatedness are estimated for a number of small-scale societies but vary widely, with mean relatedness decreasing, flat, or increasing with age, depending on immigration rates, sex-biased dispersal, and marriage residence customs (38). Therefore, we examine all three cases where mean relatedness to a given sharing group by a female age *x* is constant (the simplest case for comparison), declining (female-biased dispersal or patrilocality), and increasing (as when females remain in natal group) (see *SI Appendix*, *Additional Methodological Details and Results*).

### Selection on Production Transfers.

We estimate the force of selection on production at each age through the elasticity of fitness to production transfers. Whereas indirect fitness contributions (Δ*λ_Px_*) aggregate across all individuals of a given age class, elasticities (*e_Px_*) estimate the potential for indirect contributions as the percentage increase in focal group growth rate (fitness) that comes from a 1% increase in per capita caloric production by individuals of a given age, given mean relatedness (*r_x_*), and the degree of cooperation (*k*). Elasticities estimate the relative benefit of increasing production at different ages and can be compared with the relative benefits of increasing fertility at different ages to infer the optimal investment in either production or reproduction across the life cycle. We use a serial sensitivity tracing the linked effects of changes in age-specific production, through nutritional effects on vital rates to their inclusive fitness effects.

In addition to *r_x_* and *k*, the fitness elasticity to production involves three terms: 1) the fitness sensitivity $\left(s_{ij}=\frac{d\lambda }{da_{ij}}\right)$ of population growth (*λ*) to each vital rate *a_ij_*; 2) the response of each vital rate *a_ij_* to nutrition $\left(\frac{da_{ij}}{dE}\right),$ as reflected in the food ratio (*E*); and 3) the contribution of production at age *x* to total population production and thus to the food ratio (*E*) $\left(\frac{dE}{dP_{x}}\right).$

The total sensitivity to production at age *x* is the sum, across recipients at all ages, of all the nutritional effects stemming from production at age *x*, discounted by mean relatedness (*r_x_*) of the donor and by extent of reliable cooperation (*k*). Because survival and fertility respond differently to the food ratio (*E*), we parse fitness effects into those made by nutrition-dependent survival [*p_x_*(*E*)] vs. nutrition-dependent fertility [*m_x_*(*E*)] at each age, with a food ratio (*E*) that is affected by production at a given age *x*:

[2] $$s_{Px}=\frac{d\lambda }{dP_{x}}=r_{x}k\left(\sum_{x}\frac{\partial \lambda }{\partial p_{x}}\frac{\partial p_{x}}{\partial E}\frac{\partial E}{\partial P_{x}}+\sum_{x}\frac{\partial \lambda }{\partial m_{x}}\frac{\partial m_{x}}{\partial E}\frac{\partial E}{\partial P_{x}}\right).$$

The sensitivity of fitness to caloric production at each age (*s_Px_*) reflects selection on production, and thereby additional selection on survival, across the life cycle. To compare with selection acting on reproduction, we estimate the fitness elasticity of caloric production *e_Px_*, which scales sensitivities by mean production and by the population growth rate to estimate the percentage of change in fitness that would accrue to a 1% change in production (similar to classic elasticities estimating the relative impact of a 1% change in fertility or mortality):

[3] $$e_{P_{x}}=s_{Px}\left(P_{x}/\lambda \right).$$

Whereas fitness elasticities are traditionally scaled to sum to unity across all vital rates (survival and fertility), we scale production elasticities so that the sum across ages equals the total elasticity to fertility (about 3.5% of all vital rate elasticities in hunter-gatherers and 4.0% in chimpanzees). Although the values of direct and indirect elasticities are not directly comparable, this scaling permits comparison of the ages of peak elasticity to fertility and food production.

The second term $\left(\frac{da_{ij}}{dE}\right)$ is the response of the scalar *Z* applied to vital rates *a_ij_* (*a_ij_* = *p_x_*, *m_x_*) as the food ratio *E* changes, assuming curvature parameter (*γ*) (reflecting the slope of Eq. 1, Fig. 1*A*). Because fertility is multiplied by *Z*, the derivative of fertility (*m_x_*) with respect to the food ratio *E* is

[4] $$\frac{dm_{x}}{dE}=\frac{d}{dE}Z_{}m_{x}^{}=\frac{m_{x}^{}(\gamma +1)}{\gamma +E}\left(1-\frac{E}{\left(\gamma +E\right)}\right).$$

In contrast, mortality is divided by *Z* and survival (*p_x_*) is the complement of mortality (*p_x_* = 1 ‒ *q_x_*), so the derivative of survival with respect to the food ratio *E* is

[5] $$\frac{dp_{x}}{dE}=\frac{d}{dE}\left(1-\frac{q_{x}}{Z}\right)=\frac{q_{x}}{(\gamma +1)E}\left(\frac{\left(\gamma +E\right)}{E}-1\right).$$

The third term in Eq. **1** is the response of the food ratio *E* to production at age *x*. The population is nutritionally replete at *E* = 1 when *P_T_* = *D_T_*, but when *P_T_* < *D_T_*, then *E* < 1 and vital rates are reduced as described by Eq. **1**. Therefore, the derivative of the food ratio *E* to age-specific production is

[6] $$\frac{dE}{dP_{x}}=\frac{d}{dP_{x}}\frac{P_{T}}{D_{T}}=\frac{d}{dP_{x}}\frac{\sum_{x}P_{x}l_{x}}{D_{T}}=\frac{l_{x}}{D_{T}}.$$

Reassembling the series of responses, the derivative of population growth (*λ*) with respect to age-specific production (*P_x_*) is

[7] $$\frac{d\lambda }{dP_{x}}=r_{x}k\left(\sum_{x}s_{x+1,x}\left(\frac{q_{x}^{\left(0\right)}}{(\gamma +1)E}\left(\frac{\left(\gamma +E\right)}{E}-1\right)\right)\frac{l_{x}}{D_{T}}+\sum_{x}s_{1x}\left(\frac{m_{x}^{\left(0\right)}(\gamma +1)}{\gamma +E}\left(1-\frac{E}{\left(\gamma +E\right)}\right)\right)\frac{l_{x}}{D_{T}}\right).$$

where *s_x+1,x_* is the sensitivity to survival from age *x* to *x*+1, and s*_1x_* is the sensitivity to fertility at age *x*.

### Indirect Fitness Contributions via Production Transfers.

To estimate the indirect fitness contribution (Δ*λ_Px_*) made by production transfers contributed at each age *x*, we multiply age-specific production (*P_x_*) by the fitness sensitivity to production (*dλ*/*dP_x_*):

[8] $$\Delta \lambda_{Px}=\;\;P_{x}\left(d\lambda /dP_{x}\right).$$

Because Δ*λ_Px_* estimates the fitness that is contributed indirectly by age *x* individuals, it is equivalent to the fitness that would be lost to a population if all age *x* individuals were unable to forage for themselves (e.g., due to illness or injury).

### Indirect Contributions as Fertility Equivalence.

We estimate the equivalent number of offspring (m_x__*_) that a mother would have to produce at a given age to result in the same fitness contribution we estimate would come from their production transfers, assuming fitness sensitivities to fertility estimated at the ages of recipients. To do this for a given age, we set direct fertility contributions equal to production contributions and then solve for the fertility (m_x__*_) that would yield an equivalent contribution, where s_mx_ m_x__*_ = *s_Px_ P_x_*.

### Chimpanzees and Humans.

Female age-specific mortality and fertility rates for wild chimpanzees, hunter-gatherers, and horticulturalists come from ref. 35. Female production and consumption profiles for chimpanzees and hunter-gatherers come from ref. 2 and those for horticulturalists from ref. 4. These data were collected during periods of traditional subsistence with minimal to no medical intervention or market transactions. No living population represents an entire typology like “hunter-gatherer” or “horticulturalist,” so we also analyze data available for specific populations (*SI Appendix*, Fig. S3): Ju/’hoansi (25), Ache (89), Hadza (90), and Hiwi (91) hunter-gatherers and Yanomamo (92), Tsimane (93), Machiguenga and Piro (40), and Gainj (94) horticulturalists. Despite inhabiting diverse environments and having distinct histories, results are qualitatively similar across populations (*SI Appendix*, Fig. S9).

### Comparison of Subsistence Regime and Life History on Force of Selection.

To assess the relative influence of life history vs. subsistence regime on indirect fitness contributions and the force of selection, we compare hypothetical composites permuting the life histories and subsistence profiles of chimpanzees, human hunter-gatherers, and horticulturalists. For each permutation, we also calculate mean ages of production (*x_P_*) and demand (*x_D_*) in the same manner as mean age of reproduction [*x_M_* = (**Σ_x_** *x m_x_*)/(**Σ_x_** *m_x_*)] but with production (*P_x_*) or demand (*D_x_*) inserted instead of fertility (*m_x_*).

### PV.

To provide a prospective measure of individual fitness value comparable to Fisher’s RV (31), we calculate PV at each age. Because PV estimates the future fitness contributions expected to accrue through production transfers across the remaining lifespan, age patterns of PV and RV are directly comparable. To be directly comparable to RV, PV replaces the direct fitness contributions to the net reproductive rate *R*_0_ (*R*_0_ = **Σ_x_** *l_x_ m_x_*) with indirect contributions to *R*_0_ predicted for individuals age *x*. To do this, we compare the baseline *R*_0_^(0)^ to the *R*_0_*, the net reproductive rate calculated with *l_x_* and *m_x_* reduced by the amount predicted by Eq. **9** when production at age *x* is zero, and further discounted by population growth rate (λ), relatedness (*r_x_*) and cooperation (*k*). For each age *x* we insert this difference (Δ*R_Px_* = *R*_0_^(0)^ ‒ *R*_0_*) in place of the fertility contributions *R_x_* (*R_x_* = *l_x_ m_x_*) to estimate the prospective fitness value of future production:

[9] $${\text{PV}}_{x}=\frac{r_{x}k}{l_{x}}\sum_{y\geq x}\lambda^{-(y-x+1)}{}_{}\Delta R_{P_{y}}=\frac{r_{x}k\lambda^{\left(x-1\right)}}{l_{x}}\sum_{y\geq x}\lambda^{\left(-y\right)}\left(R_{0}^{\left(0\right)}-R_{0}^{*}(P_{y}=0)\right).$$

### Fitness Contributions of Pedagogy.

To estimate the indirect fitness contributions expected from information transfers (i.e., pedagogy), we adapt a model of optimal pedagogy from ref. 32, where social information transfers help increase the rate of skills acquisition underlying production. That model predicts the effects of information transfer on survival-discounted lifetime food production [*P_T_*(*φ*, *θ*, *a*, *b*, *t*)] given a pedagogical regime characterized by the production costs to teachers (*φ*), the boost to skills acquisition enjoyed by pupils (*θ*), the age at which teachers begin instruction (*b*), the age of pupils at the onset of instruction (*a*), and the duration of learning (*t*). Production gains to pedagogy are larger for high-skill activities, and pooled production is maximized under early-life tutoring by elders, since teachers in their prime stand to forfeit more production by teaching (32). As outlined above, we assume marginal baseline nutrition (*E* < 1, *r* = 0.01) and estimate the potential for second-order indirect fitness contributions of pedagogy investments that accrue through the nutritional effects of increased pupil production. We compare population growth rates under conditions with and without different regimes of pedagogy, as defined by (*φ*, *θ*, *a*, *b*, *t*), for a hunter-gatherer life history and production profile calibrated at the same 1% baseline.

## Supplementary Material

Supplementary File

## Data Availability

In *SI Appendix*, File S1, we provide the data used in our analyses (*SI Appendix*, File S2 is formatted for MATLAB). In *SI Appendix*, File S3, we also provide MATLAB (MathWorks, v. 2021a) code for estimating the three indicators of selection intensity (fitness contributions, elasticities, and productive value), given age profiles of survivorship (*l_x_*), fertility (*m_x_*), production (*P_x_*), nutrition curvature parameter (*γ*), and assumptions about age patterns of mean relatedness to the sharing group (*r_x_*) and reliability of cooperation (*k*). In addition, we provide a MATLAB function (*SI Appendix*, File S4) and a stand-alone application (*SI Appendix*, File S5) constructed with MATLAB Compiler. This application allows users without an active MATLAB license to replicate our results or to estimate the selection indicators for any population with appropriate data. Additional information on how to use these files is given in *SI Appendix*, Files S1–S5. Data, MATLAB code, MATLAB function, and stand-alone application for replicating analyses and accompanying information are available via the Open Science Framework (https://osf.io/ag5yp/) (95). All other study data are included in this article and/or supporting information.
