# Supplementary material for: The importance of elders: Extending Hamilton’s force of selection to include intergenerational transfers
Source: Proc Natl Acad Sci U S A. 2022 Jul 6;119(28):e2200073119. doi: 10.1073/pnas.2200073119 (PMC9282300; doi:10.1073/pnas.2200073119)
Supplement: Supplementary File [file pnas.2200073119.sapp01.pdf]

Supplemental Material for Davison and Gurven, "The importance of elders: extending Hamilton's force of selection to include intergenerational transfers"

Table of Contents

Appendix 01. Additional Methodological Details and Results
- Nutritional response of vital rates: Justification for our approach
- Comparing results with different age patterns of relatedness
- Stylized model comparing low- vs. high-skill subsistence niches

Fig. S1. Life history under food deprivation

Fig. S2. Boundary analysis

Fig. S3. Baseline life history and subsistence conditions (empirical examples).

Fig. S4. Relatedness and cooperation effects on indirect fitness contributions

Fig. S5. Effect of age patterns of relatedness.

Fig. S6. Fitness effects of production (low- vs. high-skill).

Fig. S7. Remaining production and fitness contributions.

Fig. S8. Average ages of net production and consumption.

Fig. S9. Fitness contributions, elasticities and prospective fitness value

Fig. S10. Fitness contributions by demographic regime and subsistence ecology.

Fig. S11. Required chimpanzee foraging time for 3% population growth.

Fig. S12. Average relatedness under different natal residence patterns

Table S1. Variable definitions and source equations

Table S2. Remaining direct vs. indirect fitness contributions at ages 30, 40 and 50.

Table S3. Effects of age and community characteristics on mean relatedness.

File S1. ***DATA_file.xls*** – *Excel* spreadsheet containing subsistence and life history data tables

File S2. ***DATA.csv*** – comma separated value (*.csv*) file with subsistence and life history example data (hunter-gatherers)

File S3. ***Px_file.m*** - containing *Matlab* code (*.m* file format, *Matlab* v. 2021) for all analyses, requires Matlab license

File S4. ***Px_function.m*** - *Matlab* function (*.m* file format, *Matlab* v. 2021) for analyses, requires Matlab license

File S5. ***Px_app.exe*** - stand-alone application (*.exe*) that does not require a *Matlab* license

Supplemental Material for Davison and Gurven, "The importance of elders: extending Hamilton's force of selection to include intergenerational transfers"

Appendix 01 – Additional Methodological Details and Results

Nutritional response of vital rates: Justification for our approach. While food availability clearly improves fertility and survival under marginal nutritional status, the functional relationship between human vital rates and caloric availability is poorly resolved. Many studies relate food insecurity to health and ovarian function (e.g. 1, 2, 3), or relate periods of famine or changes in food prices to macro-changes in vital rates (4, 5). Natural fertility populations experiencing undernutrition may have later age at menarche or prolonged lactational amenorrhea, thereby increasing inter-birth intervals (3). In other situations, extra food may benefit infant and child survival but not increase fertility (6). In general, population growth is more resilient to short-term changes in fertility than survival, as fertility can recover in future years whereas individuals who die forfeit all future reproduction. Thus, fitness sensitivities to survival are higher than those to fertility among many animals (7), including humans (8). Vital rate responses to nutrition are likely concave, with minor food deprivation having small costs that increase sharply with greater deficits (2, 9), an assumption supported indirectly through mortality responses to temporal changes in food prices (10, 11). Given uncertain relationships, we vary the curvature of vital rate responses to nutrition, from strong, nearly-linear responses (high γ values close to 1) to weak responses where severe deprivation is required for large effects (γ near 0). This insures that our qualitative findings are robust to a wide range of nutritional responses. Also, because we assume food-limited populations, we do not consider harmful effects of "hypernutrition" or attempt to identify the actual threshold of replete nutrition, instead assuming that "replete" nutrition would drive vital rates near the maximum observed (3% for hunter-gatherers and chimpanzees, 4% for horticulturalists, (12)) .

Comparing results with different age patterns of relatedness. Mean relatedness to the group can vary with age, but also can differ widely across societies and contexts, given patterns of sex-specific dispersal, group composition, social organization, and other socioecological factors. As our modeling exercise considers focal sharing groups among hunter-gatherers, we were primarily interested in "ground-truthing" age profiles of biological kinship in small-scale societies. To accomplish this, we take advantage of a recent paper by Koster and colleagues that compiled patterns of kinship across the life course in 19 societies varying in subsistence and demography (13), including hunter-gatherers, pastoralists and agrarian populations. Public data provided by the authors report individual-level mean biological relatedness of individuals to all other group members, for 7,434 individuals of all ages, from 19 populations in 11 countries. Relatedness comes from census and genealogical data collected by anthropologists, at a depth of at least three generations (see 13 for more details).

We first ran an analysis of average relatedness as a function of community size, which ranged from 76 to 1,387 people. We fit a power function, where avg. $r = 5.1046*(\text{community size})^{-1}$. For community sizes of 25, 50, 150, predicted average relatedness (r) is 0.20, 0.10, and 0.03, respectively. These community sizes closely mirror estimated average residential band, aggregated group, and periodic aggregation size (also Dunbar's number) for a large sample of hunter-gatherers (14).

A recent study of Agta forager kinship patterns (15) not included above shows average relatedness in Agta camps to be ~0.08, with some smaller camps showing average relatedness above 0.15 (Figure 1*C* in (15)). The average r across 34 hunter-gatherer residential groups was also reported to be 0.08 (range: 0.02 to 0.19), lower than that reported among 24 horticulturalist groups (average r = 0.11), despite the latter often living in larger populations (16). At smaller group sizes (<25 adults), however, average relatedness in hunter-gatherers is similar to that of horticulturalists (closer to r = 0.13).

While residential groups of hunter-gatherers consist of many loosely or unrelated individuals (see also 17), daily production, food sharing and other forms of cooperation are usually limited to a subset of families within the

population. *The average relatedness is thus higher within focal sharing groups consisting of common social partners giving and receiving food and other goods and services, than among the population at large.* For example, based on estimates provided by Dyble and colleagues (18), the average relatedness within sharing groups among Mbendjele BaYaka and Agta foragers is closer to 0.2 (average *r* within households is 0.33 and 0.39, respectively; average *r* beyond household to 2.7 (BaYaka) and 3.3 (Agta) other households is 0.08 and 0.10, respectively; an approximation of average *r* among all members of the sharing cluster is 0.15 and 0.18, respectively).

Nonetheless, much reliable cooperation also occurs among unrelated individuals in human subsistence societies. This has been touted as one of the hallmark achievements of the human species (19-21). Reaping the gains of cooperation and coordination while managing the "free-rider" problem is fundamental. Solutions exist in a variety of social systems combining elements of mutualism, reciprocity, needs-based sharing, and enforced social norms. Thus, even when kinship is relatively low, reliable cooperation is still possible under conditions common in human populations (indicated by higher *k* in our modeling framework). Under conditions where collective gains from mutual support or turn-taking are enforced, "group nepotism" is hypothesized to result in an ethic of unidirectional kin support (22). Taken together, it is remarkable that average kinship is lower in hunter-gatherer camps than in other highly social mammals (23). We speculate that lower kinship in hunter-gatherers might actually help limit in-group exploitation and reproductive skew, thereby supporting greater egalitarianism.

Next, we sought to determine how mean relatedness might vary by age. Koster and colleagues examined age profiles of relatedness to juveniles and adults for natal and immigrant adults in each of 19 populations (see Fig. S1 in ref. 1). Those shapes tend to be decreasing with age in 10 of the populations, increasing in 7 populations, and somewhat flat in the remaining two. To obtain a general sense of the overall pattern in the combined dataset, we ran a mixed linear regression of mean individual relatedness to the group as a function of age, sex, and whether the individual was born in the community ("natal") or was an immigrant. We also include community-level variables: community size, percentage female and male adults that were born in the community, and a random intercept term for the population. We also considered a quadratic age^2 term for non-linearity, and interacted age terms with community size, though the magnitude of these effects generate results differing little from the model restricted to a linear age term.

Model results are shown in Table S3. For a population of 25 individuals, where all adult females are natal, the average relatedness to juveniles is as high as 0.17, declining to ~0.13 by age 80 (blue curve in figure below). If instead, only half the adult males and females are natal residents, then predicted average relatedness is substantially lower, and the relationship with age is pretty flat (orange line in Fig. S12). The Agta study mentioned above shows a similar pattern of mean relatedness across the life course as the blue curve above.

For simplicity in our analysis, since all parameters are fixed in a given treatment except for age, relatedness is only a function of age. In the main text of the paper, we employ the simplest case where average relatedness does not change with age, and we vary the average relatedness between 0.2 and 0.5. In the Supplement, we consider additional scenarios where average relatedness increases or decreases with age (Fig. S5). Note that increasing relatedness will serve to increase the fitness value of transfers made by older adults.

Stylized model comparing low- vs. high-skill subsistence niches. To further explore the evolutionary path from a chimp-like ancestor with low-skill foraging niche to the complex high-skill hunter-gatherer niche with skills specialization and division of labor, we examine the role of skills-intensive foraging ecology in driving human life history evolution away from that of other primates. We do this by modeling food production as a function of skill level, and comparing selection intensity metrics where everything is held constant except for the reliance on skills for generating age-specific production.

Supplemental Material for Davison and Gurven, "The importance of elders: extending Hamilton's force of selection to include intergenerational transfers"

In the stylized model following (24), age-specific production is a joint function of skill and strength: mean daily caloric production for an individual age *x* is $P_x = S_x^{\alpha} K_x^{\beta}$, where α and β modify relative dependence of production on strength and skill.. We vary parameters to generate production profiles characterized by low- vs. high-skill dependency. Caloric demand (D_x) at each age *x* comes from age-specific consumption estimates for hunter-gatherers in (25). Assuming mean hunter-gatherer vital rates yielding a population growth rate of 1% annually (26), we use the procedures described above to estimate fitness effects for high- vs. low-skill production that are also strength-dependent (high β), leading to conservative declines in older individuals' production.

Here, we contrast populations with identical vital rates, but with subsistence regimes that differ in the skill-dependence of their foraging niche: low-skill activities increase rapidly and peak early, whereas high-skill production increases slowly to a higher peak at later ages (see Methods; Fig. 1*B*,*C* and *SI Appendix*, Fig. S1). Consistent with empirical data, we assume old-age production declines due to physical senescence (24, 27). In our stylized example, subsistence independence ($P_x > D_x$) occurs later for high-skill activities (age 22 vs. age 15 for low-skill). Our approach first considers a replete population ($E = 1$) at its maximal growth rate ($r = 3\%$ in chimpanzees and hunter-gatherers, $r = 4\%$ in horticulturalists), then reduces production proportionally at each age *x* until the growth rate (r) is diminished to 1% (at $E = E_0$), the average growth rate for contemporary hunter-gatherers (26) (*SI Appendix*, Figs. S1, S2). From this baseline life history and stylized subsistence profiles, we estimate selection on production transfers and potential indirect fitness contributions using serial sensitivities (see Materials and Methods). Total population consumption equals total production, and we assume food is shared proportional to demand D_x. Other sharing rules may be more realistic, such as those prioritizing children's needs (see *SI Appendix,* Fig. S3). By not disproportionately favoring early life, where fitness elasticities are high (8), an equal-sharing rule conservatively limits the potential fitness value of transfers. Importantly, our model assumes a steady state where individuals share at ages when they have surplus, and that they also received those benefits when dependent at earlier ages, so there is no opportunity for individual "scrounging".

Compared to low-skill production, fitness elasticity to high-skill production is lower during dependent childhood, but higher in late life when production surpluses are large. For high-skill activities, residual lifetime remaining fitness due to production (i.e. Productive Value, *PV*) is larger at all ages and *PV* exceeds Fisher's Reproductive Value (*RV*) at earlier ages, especially when nutritional responses are strong (large γ, see below). This pathway enables males and non-breeding females to provide fitness benefits without direct reproduction.

Population growth rates change the most due to prime-age production (*SI Appendix*, Figs. S4, S5). Because high-skill activities have lower early production and peak later, indirect fitness contributions are higher in late life despite fewer survivors (Fig. 2*A, B*; Fig. S6*A, B*). Fitness contributions ($\Delta\lambda_{Px}$) describe the effects on population growth rate λ of production (P_x) by individuals age *x* and depend on the force of selection on production at each age, operationalized as fitness elasticities (e_{Px}) of production (Fig. S6*C, D*). Fitness elasticities represent the proportional change in population growth due to a proportional change in production at each age. In a high-skill foraging ecology, early-life production elasticities are lower, but higher at later ages compared to a low-skill feeding niche.

Similarly, the residual fitness value of indirect contributions is reflected in Productive Value (*PV*), a direct analog of Fisher's Reproductive Value, representing the prospective fitness value of expected future production instead of future reproduction. *PV* is higher in a skills-intensive feeding niche and remains high at older ages compared to low-skills foraging (*SI Appendix*, Fig. S6*E*, *F*).

Supplemental Material for Davison and Gurven, "The importance of elders: extending Hamilton's force of selection to include intergenerational transfers"

Supplemental References

1. Scott S & Duncan CJ (2008) *Demography and nutrition: evidence from historical and contemporary populations* (John Wiley & Sons).
2. Livi-Bacci M (1990) *Population and nutrition: An essay on European demographic history* (CUP Archive).
3. Wood JW (1994) *Dynamics of Human Reproduction: Biology, Biometry and Demography* (Aldine de Gruyter, New York).
4. Chamberlain AT (2006) *Demography in archaeology* (Cambridge University Press).
5. Bengtsson T & Dribe M (2006) Deliberate control in a natural fertility population: Southern Sweden, 1766–1864. *Demography* 43(4):727-746.
6. Pennington RL (1996) Causes of early human population growth. *American Journal of Physical Anthropology* 99(2):259-274.
7. Crone EE (2001) Is survivorship a better fitness surrogate than fecundity? *Evolution* 55(12):2611-2614.
8. Jones JH (2009) The force of selection on the human life cycle. *Evolution and Human Behavior* 30(5):305-314.
9. Lee CT, Puleston CO, & Tuljapurkar S (2009) Population and prehistory III: Food-dependent demography in variable environments. *Theoretical Population Biology* 76(3):179-188.
10. Akinlo AE & Odusanya IA (2016) Effects of Food Prices on Under-five and Infant Mortality Rates in Sub-Saharan Africa. *African Journal of Economic Review* 4(1):101-112.
11. Lee H-H, Lee SA, Lim J-Y, & Park C-Y (2016) Effects of food price inflation on infant and child mortality in developing countries. *The European journal of health economics* 17(5):535-551.
12. Davison R & Gurven M (2021) Human uniqueness? Life history diversity among small-scale societies and chimpanzees. *PLoS ONE* 16(2):e0239170.
13. Koster J*, et al.* (2019) Kinship ties across the lifespan in human communities. *Philosophical Transactions of the Royal Society B* 374(1780):20180069.
14. Hamilton MJ, Milne BT, Walker RS, Burger O, & Brown JH (2007) The complex structure of hunter-gatherer social networks. *Proceedings of the Royal Society of London, Series B* 274:2195-2202.
15. Dyble M, Migliano AB, Page AE, & Smith D (2021) Relatedness within and between Agta residential groups. *Evolutionary Human Sciences* 3.
16. Walker RS (2014) Amazonian horticulturalists live in larger, more related groups than hunter–gatherers. *Evolution and Human Behavior* 35(5):384-388.
17. Hill KR*, et al.* (2011) Co-residence patterns in hunter-gatherer societies show unique human social structure. *Science* 331(6022):1286-1289.
18. Dyble M*, et al.* (2016) Networks of food sharing reveal the functional significance of multilevel sociality in two hunter-gatherer groups. *Current Biology* 26(15):2017-2021.
19. Henrich N & Henrich J (2007) *Why humans cooperate* (Oxford University Press, Oxford).
20. Hill K, Barton M, & Hurtado AM (2009) The emergence of human uniqueness: Characters underlying behavioral modernity. *Evolutionary Anthropology: Issues, News, and Reviews* 18(5):187-200.
21. Jaeggi AV & Gurven M (2013) Natural cooperators: food sharing in humans and other primates. *Evolutionary Anthropology: Issues, News, and Reviews* 22(4):186-195.
22. Jones D (2000) Group nepotism and human kinship. *Current Anthropology* 41(5):779-809.
23. Dyble M & Clutton-Brock TH (2020) Contrasts in kinship structure in mammalian societies. *Behavioral Ecology* 31(4):971-977.
24. Gurven MD & Kaplan HS (2006) Determinants of time allocation to production across the lifespan among the Machiguenga and Piro Indians of Peru. *Human Nature* 17(1):1-49.
25. Kaplan H, Hill K, Lancaster JB, & Hurtado AM (2000) A theory of human life history evolution: Diet, intelligence, and longevity. *Evolutionary Anthropology* 9(4):156-185.
26. Gurven MD & Davison RJ (2019) Periodic catastrophes over human evolutionary history are necessary to explain the forager population paradox. *Proceedings of the National Academy of Sciences*:201902406.
27. Koster J*, et al.* (2020) The life history of human foraging: Cross-cultural and individual variation. *Science Advances* 6(26):eaax9070.

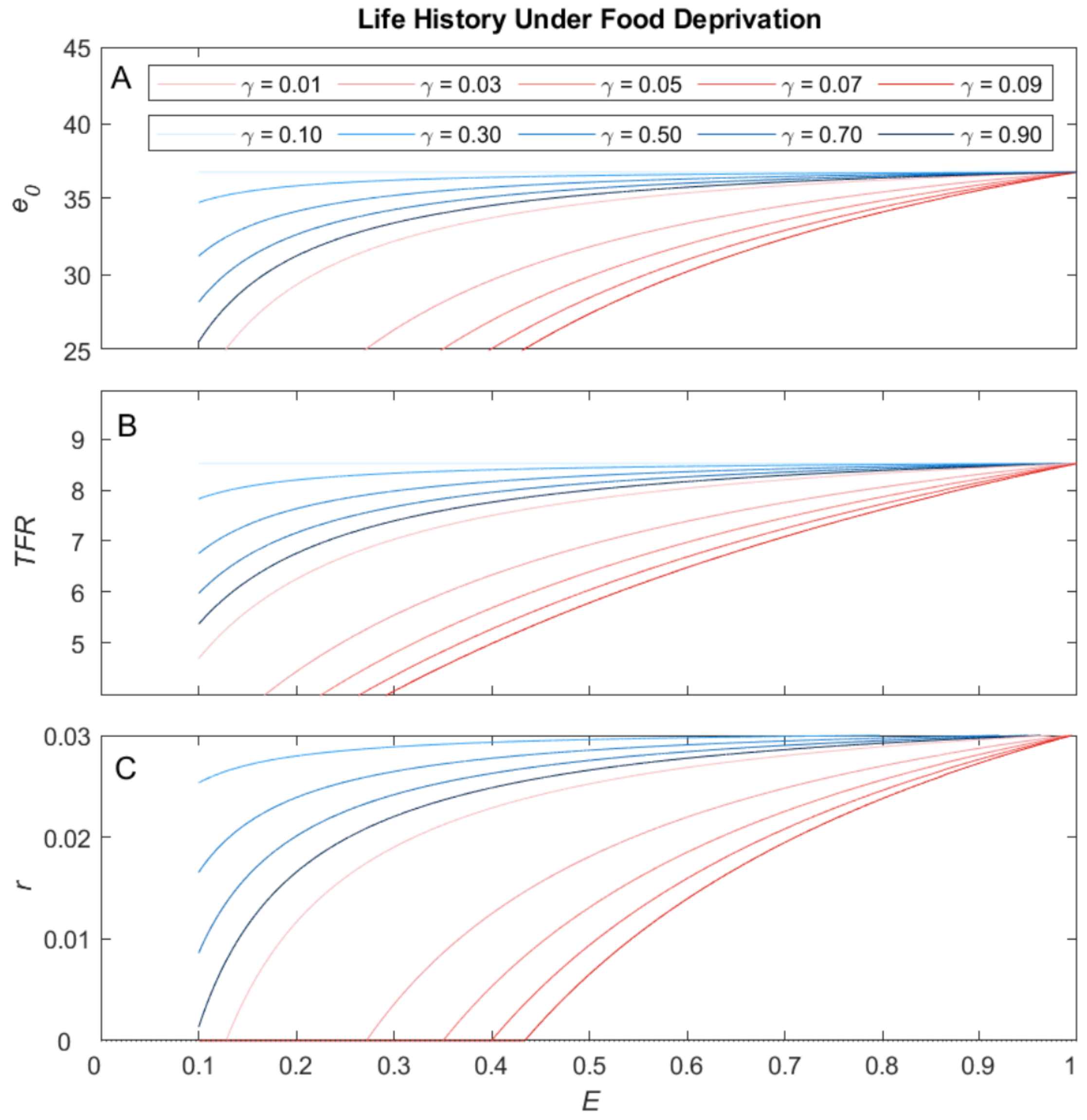

Fig. S1. Life history under food deprivation. For the average hunter-gatherer life history, panels show the effect of the Food Ratio (*E*, *x*-axes) on: (A) life expectancy (e_0, the average number of years lived from birth); (*B*) total fertility rate (*TFR*, average number of births a woman can expect to have over her lifetime if she survives throughout her reproductive years); (*C*) the exponential population growth rate (*r*, the annual rate of increase). Lines show predictions under different starvation responses (γ, in legend).

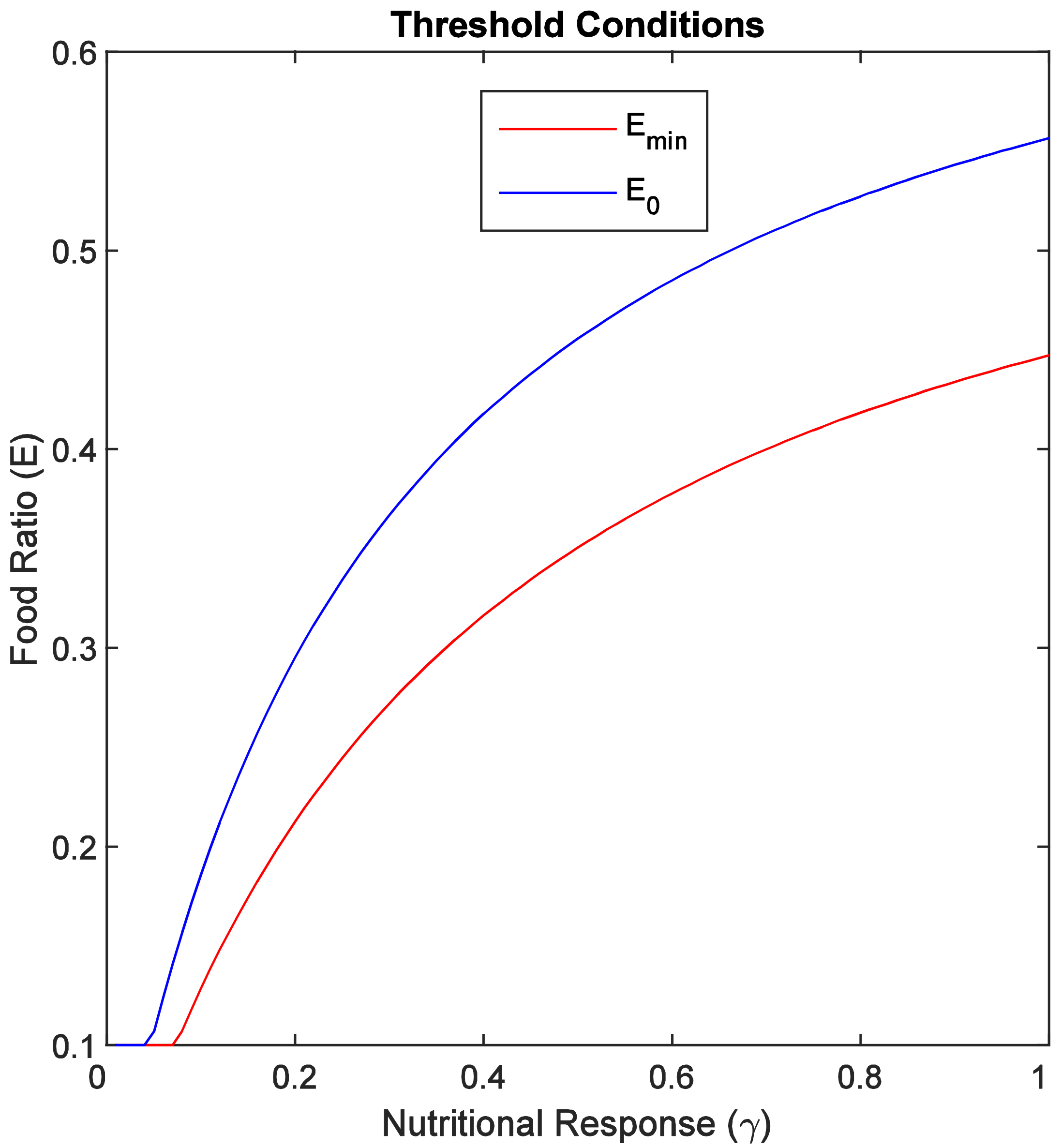

Fig. S2. Boundary analysis. Minimum food ratio (E_{min}) for stationary population growth ($\lambda = 1$) and the baseline food ratio (E_0 such that $\lambda(E_0) = \lambda^{(0)} = 0.01$) depend on the curvature of the starvation response (γ, x-axis)

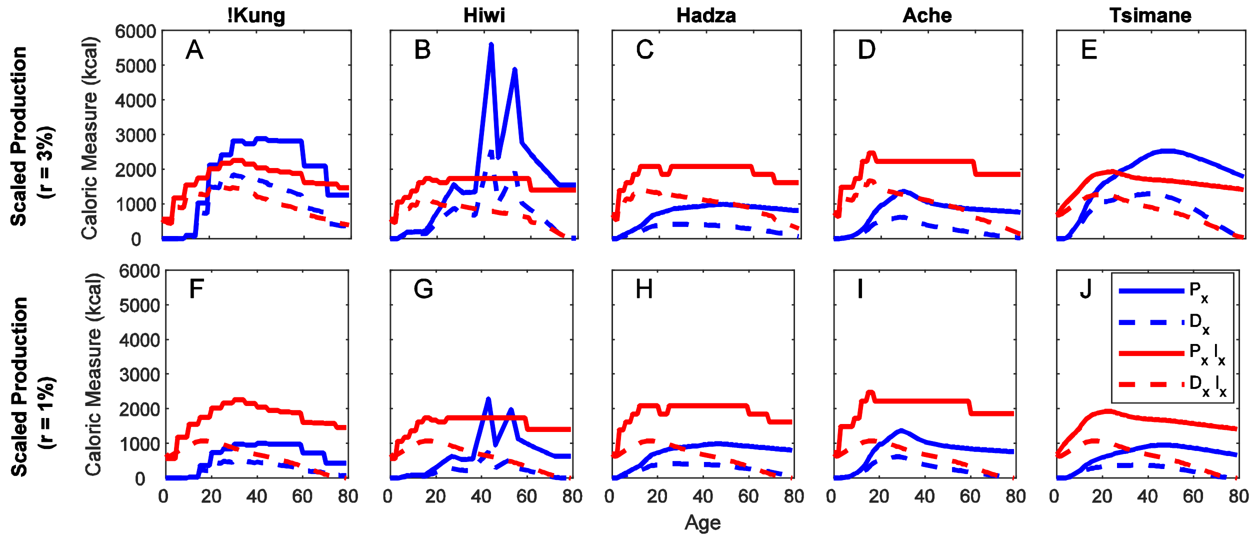

Fig. S3. Baseline life history and subsistence conditions (empirical examples). For each population, panels show age profiles of individual production (P_x, blue) caloric demand (D_x, red). Dashed lines show survival-discounted population production ($P_x l_x$) and demand ($D_x l_x$) reflecting mortality attrition driving the age structure. (*A*-*E*) Top row shows predictions for replete nutrition (E = 1; m_x and p_x~ max, r = 0.03); (*F*-*J*) bottom row shows predictions for marginal nutrition (E_0 < 1, r = 0.01) representing baseline initial conditions for production manipulations.

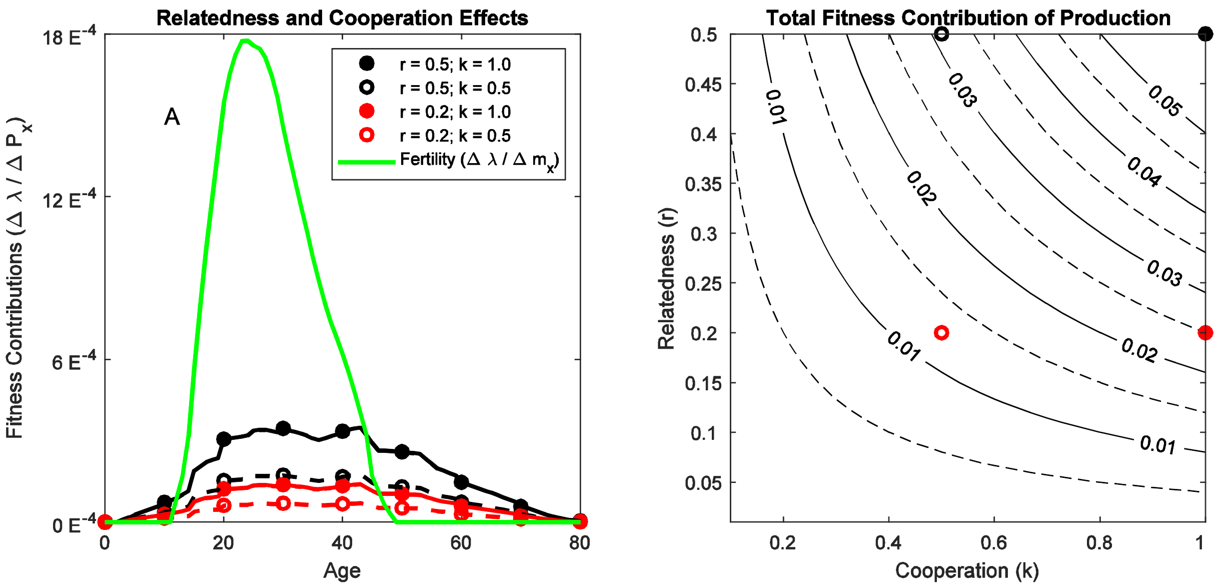

Fig. S4. Relatedness and cooperation effects on indirect fitness contributions. (A) Lines predict indirect fitness contributions ($\Delta\lambda$ / ΔP_x) for the average hunter-gatherer life history and subsistence profile under different mean relatedness (r_x) and cooperation (k), assuming moderate starvation response (γ = 0.3). Green line shows direct fitness contribution of fertility ($\Delta\lambda$ / Δm_x). (B) Total lifetime fitness contribution via production transfers ($\mathbf{\Sigma_x}$ $\Delta\lambda$ / ΔP_x) is predicted under different assumptions about mean relatedness (r_x, y-axis) and cooperation (k, x-axis). Circles indicate combinations illustrated in (A).

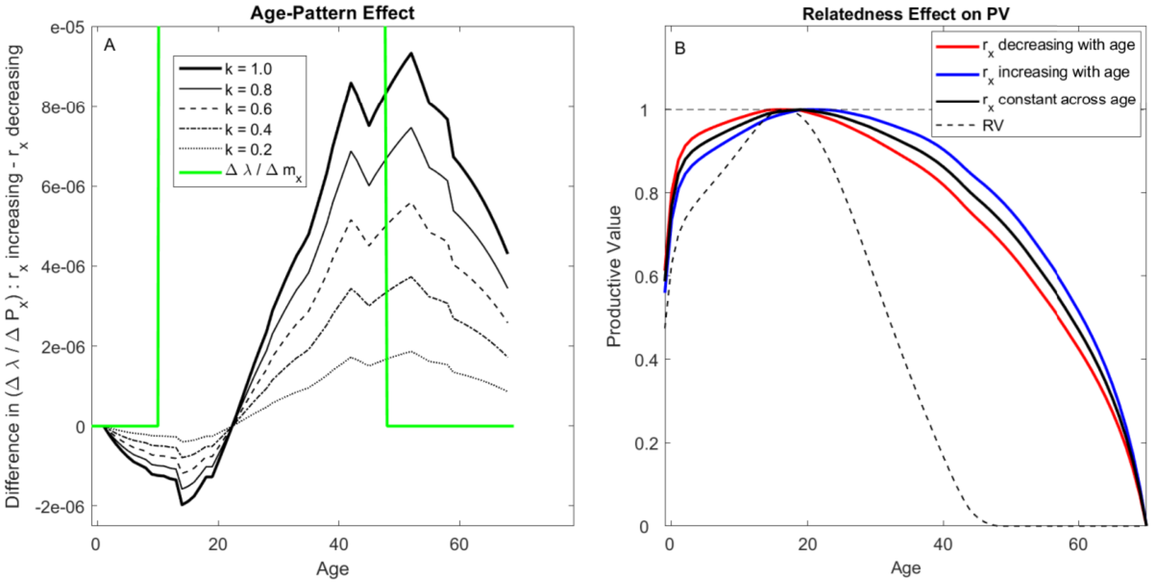

Fig. S5. Effect of age patterns of relatedness. (A) Net change in indirect fitness contributions from a scenario where mean kinship (r_x) increases with age and where mean kinship decreases with age. These are shown under varying degrees of mean cooperativeness, *k*. (B) Productive Value (*PV*) is compared between scenarios where relatedness (r_x) to the focal group either decreases, increases, or is constant across age (*x*).

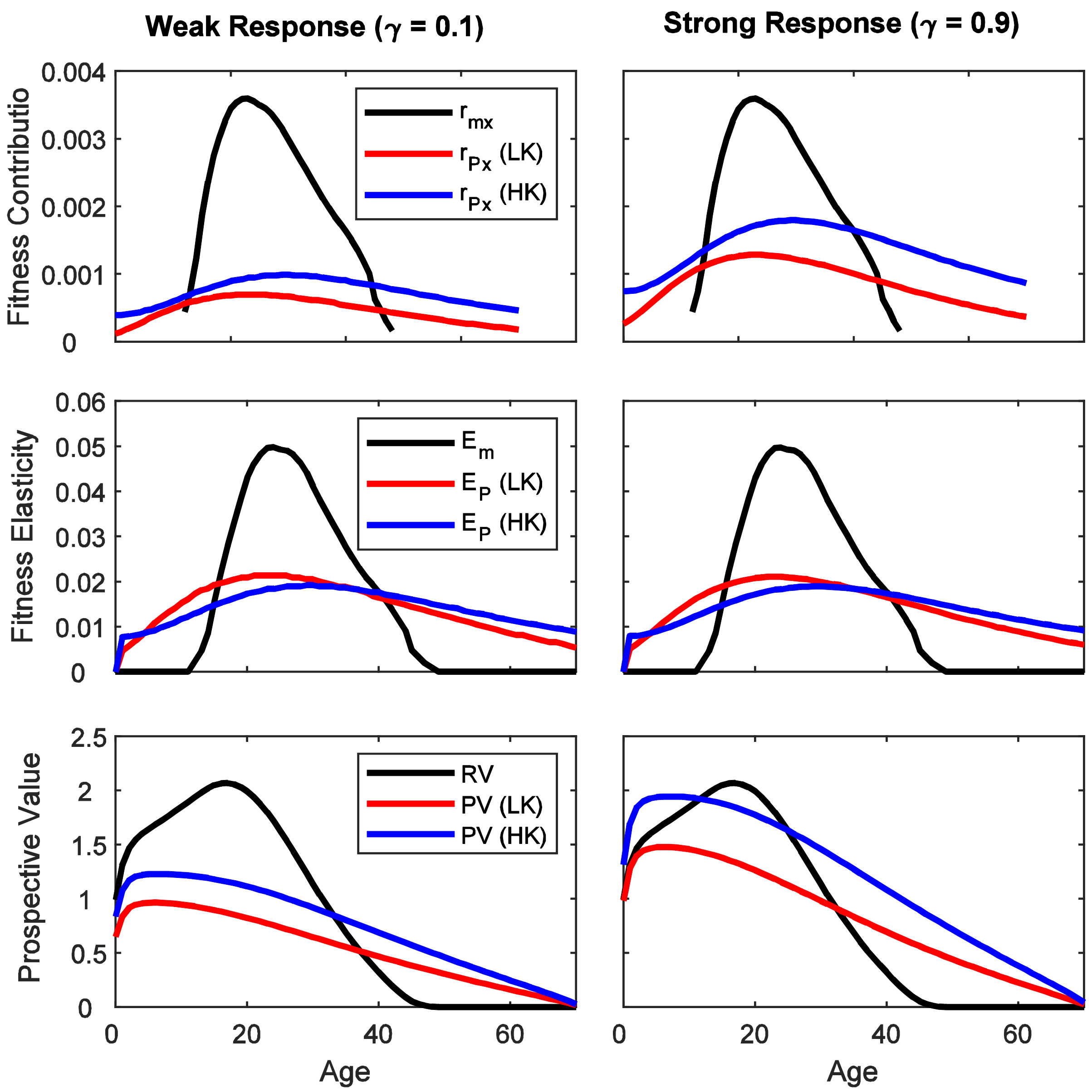

Fig. S6. Fitness effects of production (low- vs. high-skill). Comparing direct reproductive fitness (black lines) vs. indirect fitness contributions of low-skill production (red lines) or high-skill production (blue lines): (*A, B*) fitness contributions ($\Delta\lambda_{mx}$ vs. $\Delta\lambda_{Px}$); (*C, D*) fitness elasticities (e_{mx} vs. e_{Px}); (*E, F*) Reproductive Value (*RV*) vs. Productive Value (*PV*, residual future fitness effects due to expected remaining surplus production transfers). Columns compare effects of (*A, C, E*) for low-value transfers (γ = 0.1) vs (*B, D, F*) high-value transfers (γ = 0.9).

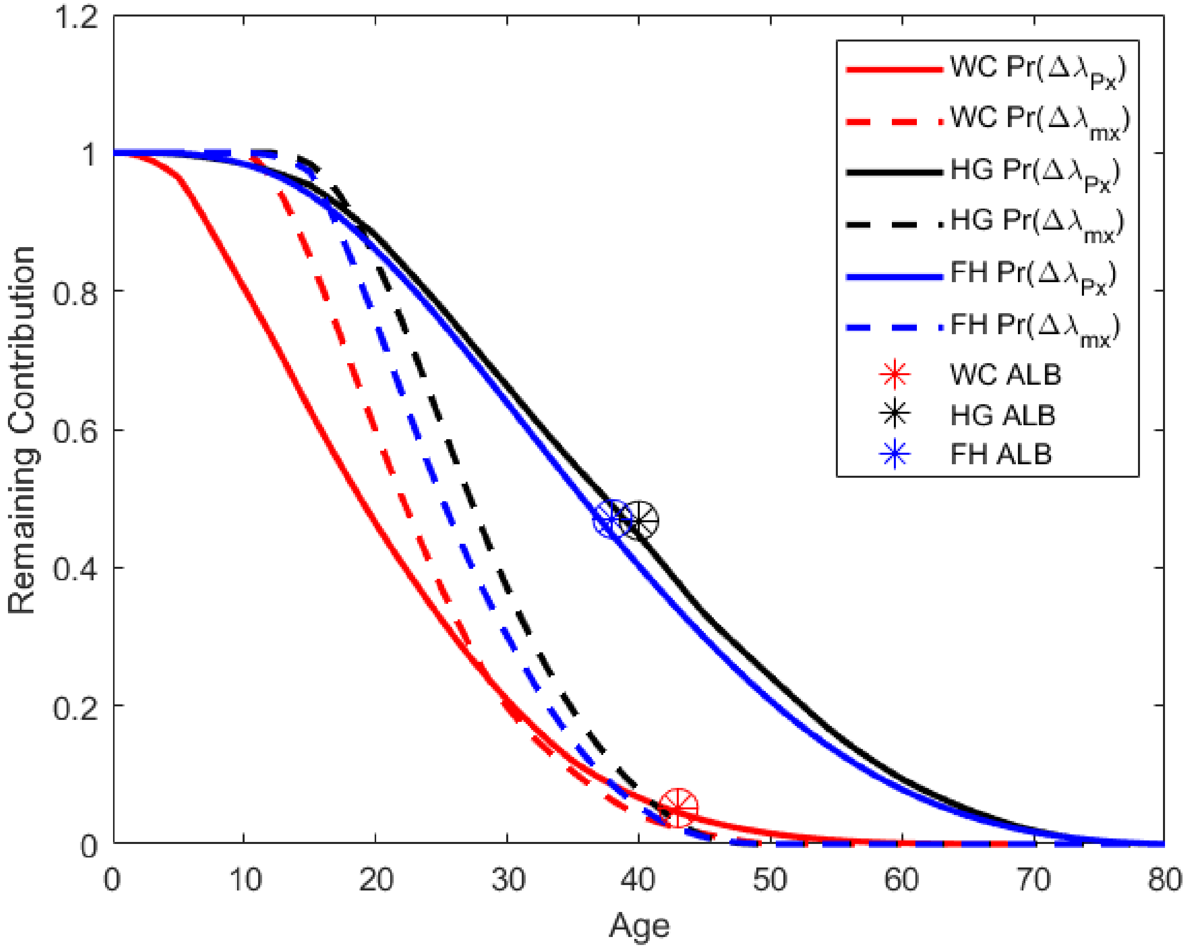

Fig. S7. Remaining production and fitness contributions. For chimpanzees (red), hunter-gatherers (black) and forager-horticulturalists (blue), solid lines show the proportion ($Pr(\Delta\lambda_{Px})$) of indirect fitness contributions ($Pr(\Delta\lambda_{Px}) = \mathbf{\Sigma_y}\ \Delta\lambda_{Py\geq x} / \mathbf{\Sigma_x}\ \Delta\lambda_{Px}$) and dashed lines in each panel show the proportion of lifetime reproduction remaining at each age ($Pr(\Delta\lambda_{mx}) = \mathbf{\Sigma_y}\ m_{y\geq x}\, l_{y\geq x} / R_0$; $R_0 = \mathbf{\Sigma_x}\ m_x\, l_x$). Circled asterisks indicate mean age at last reproduction (*ALB*).

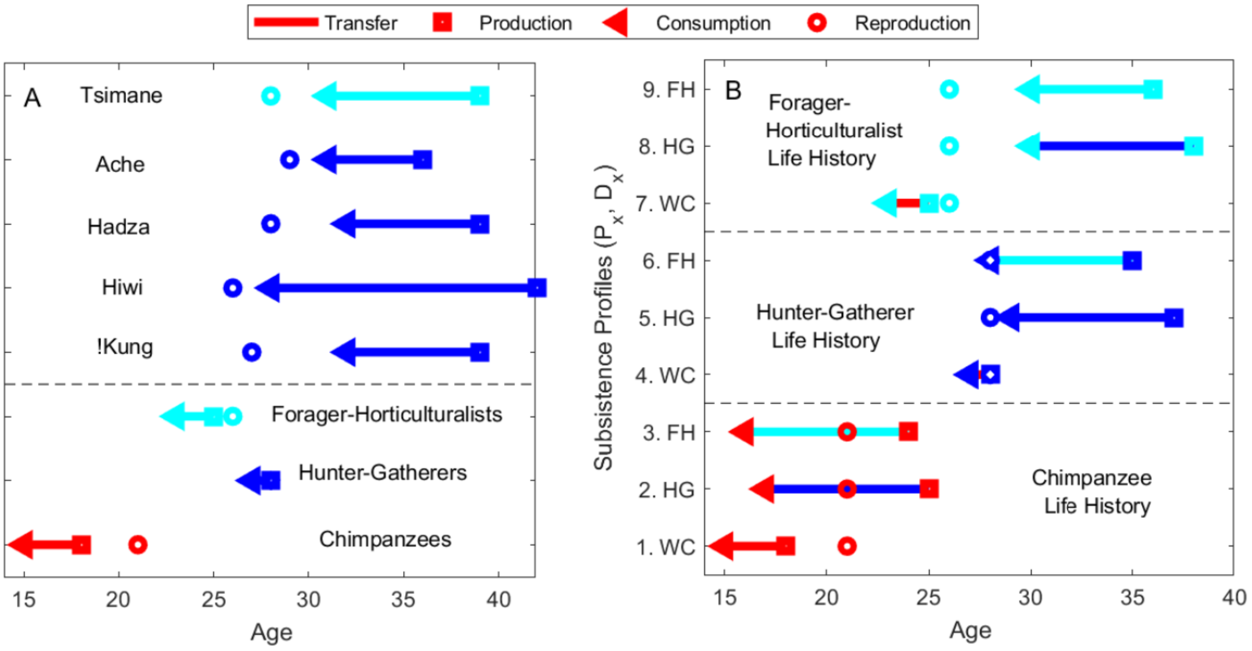

Fig. S8. Average ages of net production and consumption. (*A*) Net production transfers, as represented by the arrow connecting the average age of production (square) to the average age of consumption (arrow), for human hunter-gatherers (blue), forager-horticulturalists (teal) and chimpanzees (red). Chimpanzees transfer across a narrow range of young ages, and both mean ages of production (age 18) and demand (age 15) are younger than the mean age at childbearing (21 years). This suggests that even if chimpanzees managed to pool population production and share like hunter-gatherers, it would be mainly helping prime-age mothers feeding younger mothers, due to mortality attrition and low production surpluses. In contrast, hunter-gatherers transfer across the widest age range (age 37 to age 29), with the average-aged (28 year-old) mother receiving benefits from older individuals. Stronger demand from children and later peak production of foraging-horticulturalists drive transfers across a narrower age range (age 36 to age 30), at older ages than their earlier mean age at childbearing (age 26). (*B*) Net transfers are shown for life history x subsistence permutations across chimpanzees (red), hunter-gatherers (blue) and forager-horticulturalists (teal).

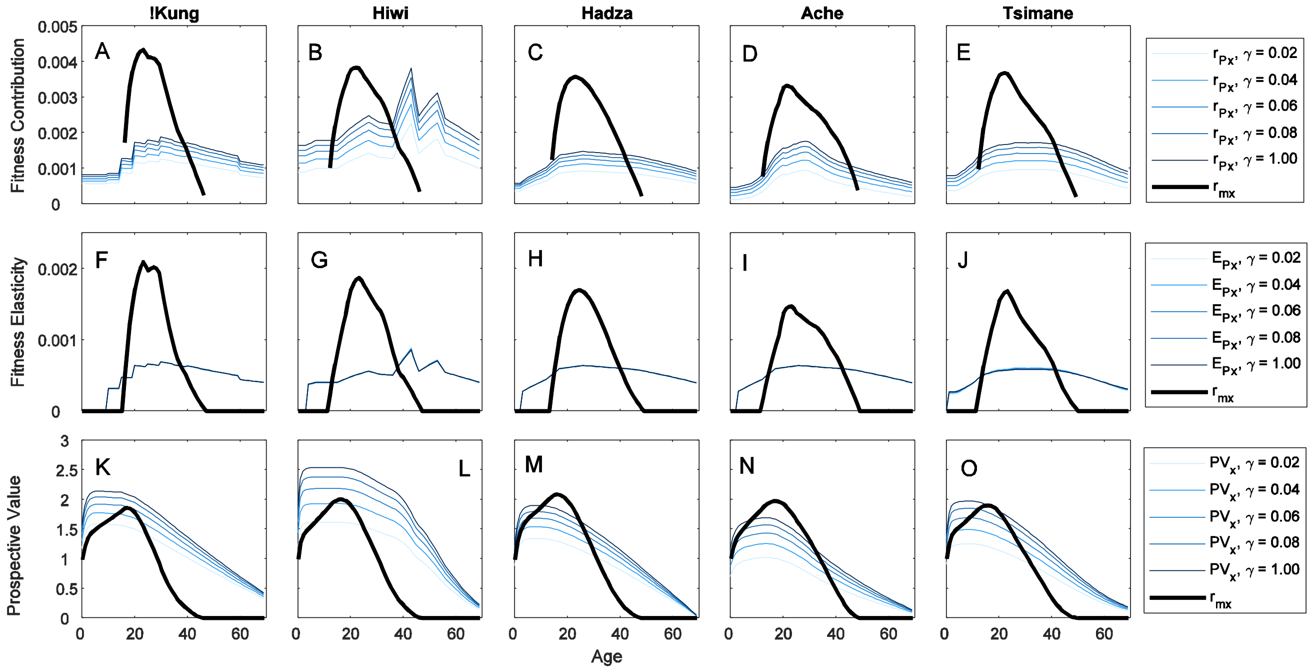

Fig. S9. Fitness contributions, elasticities and prospective fitness value. (*A*-*E*) Direct fitness contributions ($\Delta\lambda_{mx}$, black lines) to the asymptotic population growth rate (λ) vs. potential indirect fitness contributions via production transfers ($\Delta\lambda_{Px}$, blue lines show predictions under different nutritional responses (γ = 0.1 to 0.9)). (*F*-*J*) Fitness elasticities to production vs. reproduction (fertility), scaled such that the sum of fertility elasticity equals the sum of production elasticity. (*K*-*O*) Residual fitness value: Reproductive Value (black lines) vs. Productive Value (blue lines).

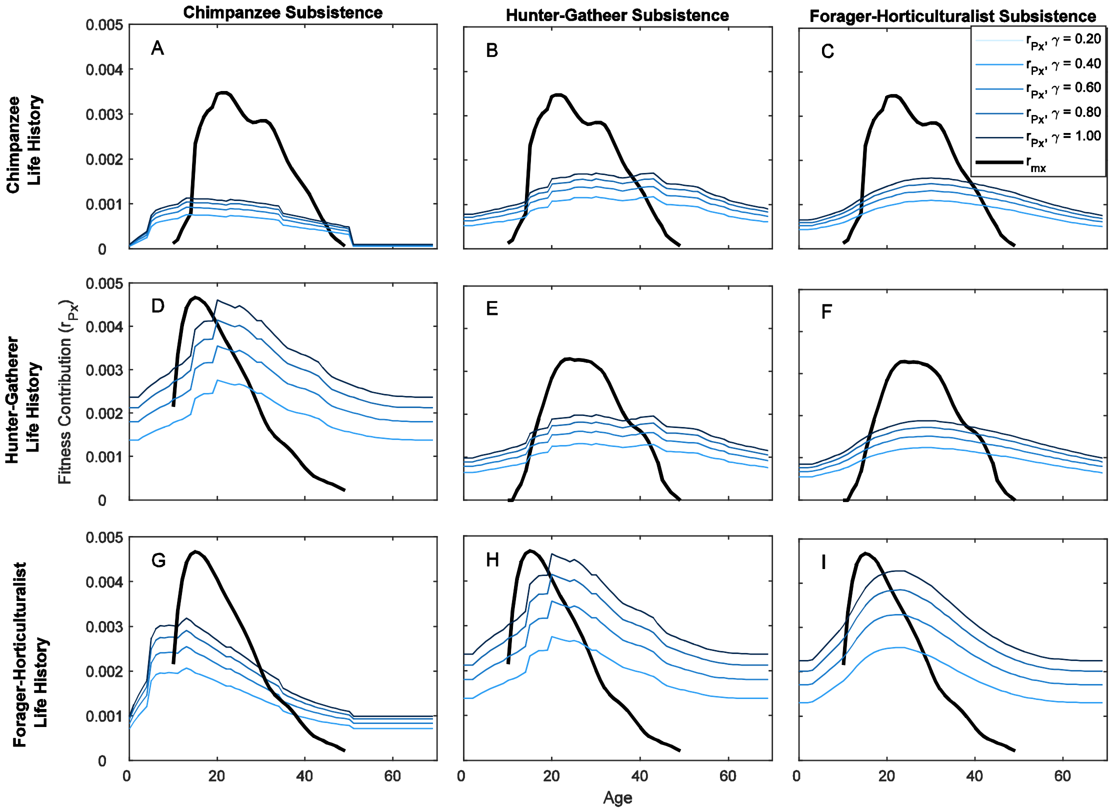

Fig. S10. **Fitness contributions by demographic regime and subsistence ecology.** Demographic regime reflects fertility and survivorship schedules of wild chimpanzees, human hunter-gatherers and horticulturalists. Subsistence ecology reflects age schedules of production and consumption for these same composite groups. Panels compare direct fitness contributions ($\Delta\lambda_{mx}$), black lines) to the population growth rate rate (*r*) vs. potential indirect fitness contributions via production transfers ($\Delta\lambda_{Px}$); colored lines show predictions under different nutritional responses, γ). The diagonal panels show predictions for (*A*) chimpanzees, (*E*) hunter-gatherers or (*I*) forager-horticulturalist, while the off-diagonal panels predict fitness contributions under different pairwise combinations of subsistence profiles (columns) and life history schedules (rows).

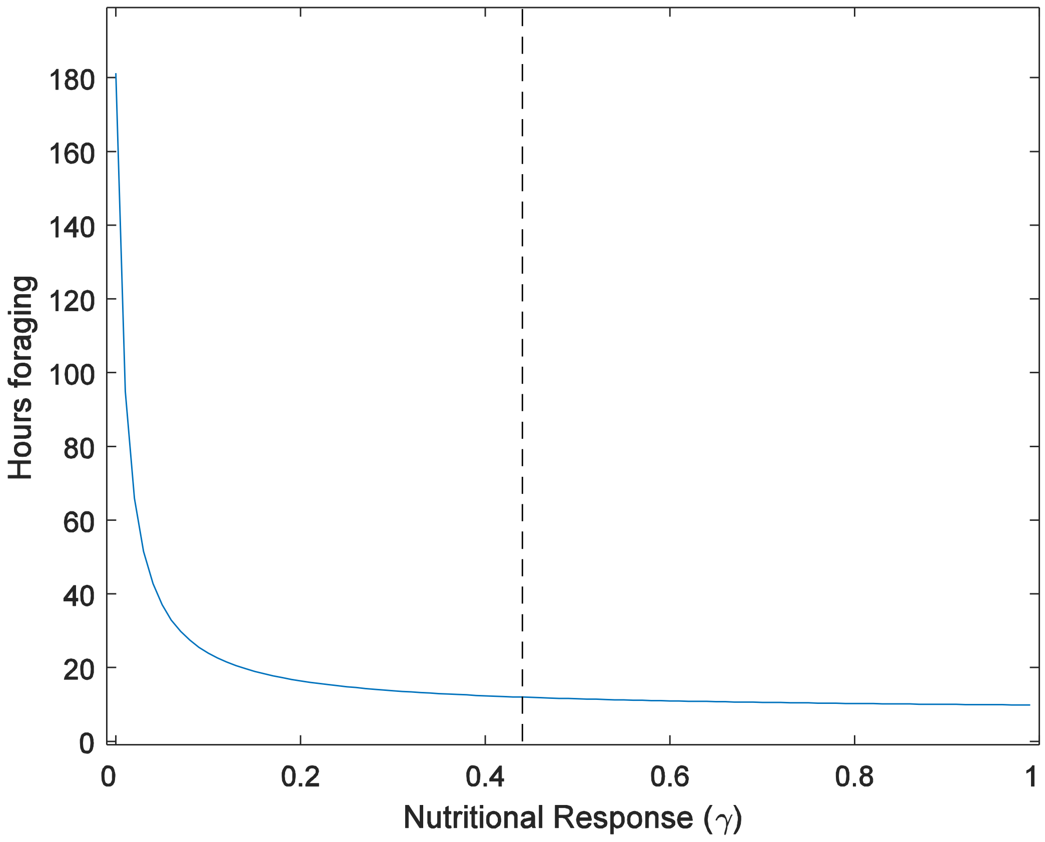

Fig. S11. Required chimpanzee foraging time to attain 3% population growth. Chimpanzees in the wild are estimated to forage for 5.63 hrs/day, at a caloric production rate of 277 kcals/hr [Kraft et al. in press]. If we conservatively assume that this level of work effort and efficiency corresponds to a population growth rate of log λ = 1%, we can estimate the additional work effort that would be necessary for adult foragers at the same chimpanzee production rate to reach a population growth rate of log λ = 3%. Daily required work effort depends on the nutritional response, γ. Dashed vertical line at 12 hrs/day corresponds to γ = 0.43. Weak effects of transfers on vital rates lead to impossibly large daily work effort requirements.

Fig. S12. Average relatedness under different natal residence patterns. Compares results for groups with 50% male and 50% females natal to group vs. 100% females and 0% males natal to group.

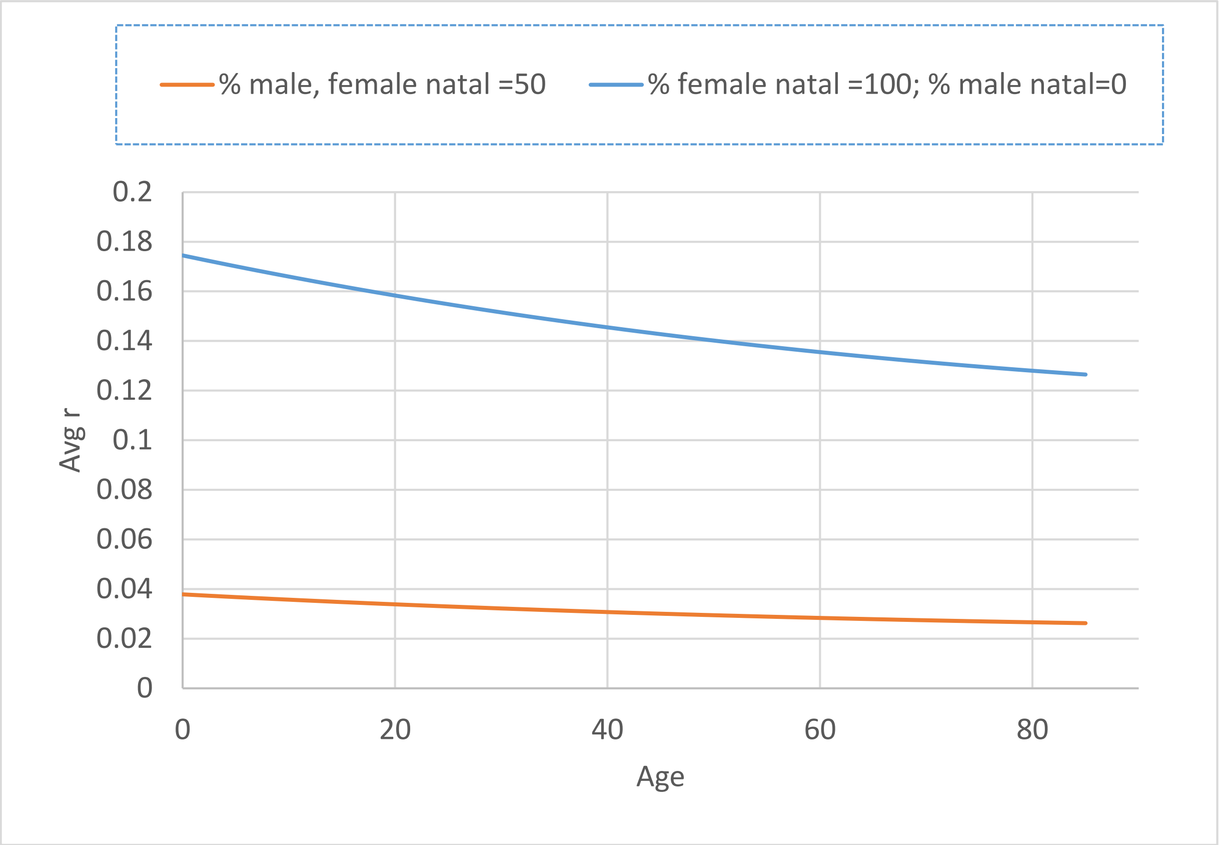

Supplemental Material for Davison and Gurven, "The importance of elders: extending Hamilton's force of selection to include intergenerational transfers"

Table S1. Definitions for all variables mentioned in the text.

Symbol	Description	Units/Values
$\mathbf{A}$	Population Projection Matrix	$\mathbf{A} = \{a_{ij}\}$
a	Age of Instruction (onset)	years
a_{ij}	Matrix Element	survival p_x, fertility m_x
b	Age of Instruction (end)	years
D_T	Demand (Total)	kcal per day
D_x	Daily caloric demand at age x	kcal per day
E_0	Baseline Food Ratio	% Replete kcal
e_{Px}	Elasticity to production at age x	% $\Delta\lambda$ per % ΔP_x
E_x	Food Ratio at age x	% demand met
K_x	Skill at age x	0-1 (% max skill)
k	Cooperation coefficient	0-1 (% cooperation)
l_x	Survivorship to age x	survival from birth to age x
m_x	Fertility at age x	daughters/mother age x
m_{x*}	Equivalent fertility at age x	daughters/mother age x
P_T	Production (Total)	kcal per day
PV_x	Productive Value at age x	residual fitness (R_0 units)
p_x	Survival from age x to x+1	Pr(survival x to x+1)
P_x	Daily production at age x	kcal produced per day
q_x	Mortality at age x	Pr(die between x and x+1)
r	Intrinsic (exponential) Growth Rate	annual pop. growth (r >/< 0)
R_0	Net Reproductive Rate (NRR)	growth rate/generation (R_0 >/< 1)
R_0*	Baseline NRR	growth rate/generation
RV_x	Reproductive Value at age x	residual fitness (R_0 units)
R_x	Fitness Contribution (to NRR) at age x	Fitness contribution (R_0 units)
r_x	Relatedness coefficient for age x female	0-1 (0.5 for direct offspring)
s_{ij}	Vital Rate Sensitivity	fitness response ($\Delta\lambda$ / Δa_{ij})
s_{Px}	Production Sensitivity at age x	fitness response ($\Delta\lambda$ / ΔP_{ix})
S_x	Strength at age x	0-1 (% max strength)
t	Duration of Instruction	years
x	Age	years
Z	Vital Rate Scalar	0-1 (% max rate)
α	Strength Effect	exponent
β	Skill Effect	exponent
γ	Starvation Response	curvature parameter
ΔR_{Px}	Change in *NRR* Due to Transfers at age x	growth rate/generation (ΔR_0 / ΔP_x)
$\Delta\lambda_{Px}$	Production contribution to fitness at age x	population growth rate ($\Delta\lambda$ / ΔP_x)
θ	Pupil Boost	scalar (0 to 1)
λ	Population Growth Rate	annual multiplier
ϕ	Teacher Cost	scalar (0 to 1)

Table S2. Residual fitness contributions at ages 30, 40 and 50. For wild chimpanzees (WC), hunter-gatherers (HG) and forager-horticulturalists (FH), metrics represent the percentage of lifetime fitness contributions remaining at three ages (30, 40 and 40 years). Direct fitness contributions reflect fertility contributions to population growth and indirect contributions estimate the inclusive fitness effects due to production at each age.

	Direct Fitness			Indirect Fitness		
**Age (y)**	30	40	50	30	40	50
**WC (%)**	20	4	0	21	7	2
**HG (%)**	37	8	0	66	45	24
**FH (%)**	30	5	0	64	40	21

Table S3. Effects of age and community characteristics on mean relatedness.

Effect	Estimate	Std Error	df	Pr > |t|
Age (y)	-0.0065	0.0018	7271	0.0003
Age*Age	0.0000	0.0000	7271	0.3392
Community size	-0.0014	0.0000	7271	<.0001
Age*Community size	0.0000	0.0000	7271	0.0006
Age*Age*Community Size	0.0000	0.0000	7271	<.0001
Sex (1=male)	-0.0146	0.0125	7271	0.2419
Natally born (1=yes)	0.0381	0.0166	7271	0.0217
% natal female	0.0193	0.0003	7271	<.0001
% natal male	-0.0144	0.0003	7271	<.0001

Supplemental Material for Davison and Gurven, "The importance of elders: extending Hamilton's force of selection to include intergenerational transfers"

File S1. ***DATA_file.xls*** – available on *Open Science Framework* (*OSF*)*;* https://osf.io/ag5yp/

Excel spreadsheet containing subsistence and life history data tables. Rows for each age with columns showing (1) age, (2) production (P_x) in kcal/day, (3) demand (D_x) in kcal/day, (4) fertility (m_x) in daughters per year, (5) annual survival probabilities (p_x). Separate worksheets show data averaged over (1) wild chimpanzees, (2) hunter-gatherers, and (3) forager-horticulturalists.

File S2. DATA.csv – available on *Open Science Framework* (*OSF*)*; https://osf.io/ag5yp/*

Comma separated value (*.csv*) file with subsistence and life history example data (hunter-gatherers). Data format is the same as in File S1: rows for each age with columns showing (1) age, (2) production (P_x) in kcal/day, (3) demand (D_x) in kcal/day, (4) fertility (m_x) in daughters per year, (5) annual survival probabilities (p_x).

File S3 – *Matlab* code ('*PV_file.m*') – available on *Open Science Framework* (*OSF*); https://osf.io/ag5yp/

Calculates all measures in study, including intermediate variables, writes three *.csv* files:

Px_contribs.csv, indirect contributions made via production transfers ($\Delta\lambda_{Px}$)
Px_elast.csv, elasticity to production (e_{Px})
Px_PV.csv, Productive Value (*PV*)

Requires manually INPUT before running:

1) *approach* (1 = simple 2D output using input r_x, k; 2 = detailed 4D output toggling across r_x and k values
2D = (*xmax* x 101); rows = age (x), columns = nutritional response (γ = 0:0.01:1);
4D = (*xmax* x 101 x 50 x 10); rows = age (x), columns = nutritional response (γ = 0:0.01:1), replicated across 50 r_x increments (r_x = 0:0.01:0.5) and 10 k increments (k = 0:0.1:1);
Note: 4D output must be reconcatenated from 2D *.csv* files
2) *rmax* (maximum population growth rate used to calibrate replete nutrition)
3) *xmax* (maximum age modeled)
4) *rx* (mean relatedness among focal sharing group)
5) *k* (mean cooperation)

File S4 – *Matlab* function ('*PV_function.m*') – available on *Open Science Framework* (*OSF*); https://osf.io/ag5yp/

Calculates all measures in study, including intermediate variables, writes three *.csv* files:
Px_contribs.csv, indirect contributions made via production transfers ($\Delta\lambda_{Px}$)
Px_elast.csv, elasticity to production (e_{Px})
Px_PV.csv, Productive Value (*PV*)

Requires INPUT when calling function by typing:

"PV_function(*approach, rmax, xmax, rx, k*)", includes 5 INPUTS separated by commas:

1) *approach* (1 = simple 2D output using input r_x, k; 2 = detailed 4D output toggling across r_x and k values
2D = (xmax x 101); rows = age (x), columns = nutritional response (γ = 0:0.01:1);
4D = (xmax x 101 x 50 x 10); rows = age (x), columns = nutritional response (γ = 0:0.01:1), replicated across 50 r_x increments (r_x = 0:0.01:0.5) and 10 k increments (k = 0:0.1:1);
Note: 4D output must be reconcatenated from 2D *.csv* files
2) *rmax* (maximum population growth rate used to calibrate replete nutrition)
3) *xmax* (maximum age modeled)
4) *rx* (mean relatedness among focal sharing group)
5) *k* (mean cooperation)

File S5 - Stand-alone application for replicating analyses – accompanying information

Available on *Open Science Framework* (*OSF*); *https://osf.io/ag5yp/*

We include a stand-alone application for reconstructing the analyses contained in this paper or applying the methods described to other data, without requiring any version of *Matlab* license. The application (***'Px_app.exe'***, *SI Appendix* File 5) constructed in *Matlab Compiler* (v. 2021a) and can be run in the Windows command prompt console (*cmd*).

Operation requires 1) A data input file in *.csv* format with columns arranged as they appear in the sample '***DATA.csv'*** provided in the *SI Appendix* (File S2) and 2) five INPUTS when calling function.

To operate in the command prompt (*cmd*), type:

"PV_app [*approach*] [*rmax*] [*xmax*] [*rx*] [*k*]", with 5 numerical INPUTS separated by spaces (not commas):

1. *approach* (1 = simple 2D output using input r_x, k; 2 = detailed 4D output toggling across r_x and k values
   2D = (*xmax* x 101); rows = age (x), columns = nutritional response (γ = 0:0.01:1);
   4D = (xmax x 101 x 50 x 10); rows = age (x), columns = nutritional response (γ = 0:0.01:1), replicated across 50 r_x increments (r_x = 0:0.01:0.5) and 10 k increments (k = 0:0.1:1);
   Note: 4D output must be reconcatenated from 2D *.csv* files
2. *rmax* (maximum population growth rate used to calibrate replete nutrition)
3. *xmax* (maximum age modeled)
4. *rx* (mean relatedness among focal sharing group)
5. *k* (mean cooperation)

Data must be arranged in rows by one-year age categories, with columns containing values for:

1. age (x) ranging from age zero newborns (x = 0) to the maximum age in the data (x = *xmax*);
2. age profiles of caloric production (P_x, gross foraging returns);
3. age profiles of caloric demand (D_x, consumption at replete nutrition where E = 1 and r = *rmax*);
4. age schedules of survival (p_x, annual survival probabilities from age x to age x+1);
5. age schedules of fertility (m_x, female offspring per mother age x).
6. age profiles of mean relatedness to the focal sharing group (identical values if constant across age)

To run properly, the data file must be named **DATA.csv** and must be saved in the same directory as the application file ('***PV_app.exe'***).

To run the application,

1. Save '***DATA.csv'*** data file to same directory as '***PV_app.exe'***
2. Open windows command (*cmd*) prompt console
3. Navigate command prompt to directory containing '***PV_app.exe***' and '***DATA.csv'***
4. Call the application file ('***PV_app.exe'***) by typing the filename followed by a space, the input values for *approach*, *rmax*, *xmax*, *rx* and *k* with spaces between (not commas).

To generate the simple (2D output) for the hunter-gatherer case included as an example data file ('***DATA.csv'***), *approach* = 1, *rmax* = 0.03, *xmax* = 71, *rx* = 0.2, *k* = 0.5, so you would type:

"PV_app 1 0.03 71 0.2 0.5"

To generate the detailed (4D output) for the hunter-gatherer case included as an example data file ('***DATA.csv***'), *approach* = 2, *rmax* = 0.03, *xmax* = 71, *rx* = 0.2, *k* = 0.5, so you would type:

"PV_app 2 0.03 71 0.2 0.5"

Results are output and saved in the same file director as three *.csv* tables (2D for simple *approach* = 1, 4D for detailed *approach* = 2):

1. **'*Px_contribs.csv*'** contains the age-specific indirect fitness contributions ($\Delta\lambda_{Px}$) made by production at each age *x*
2. **'*Px_elast.csv*'** contains the elasticities (e_{Px}) of fitness to production at each age *x*
3. **'*Px_PV.csv*'** contains the Productive Value (*PV*) estimates reflecting expected future indirect fitness contributions ($\Delta\lambda_{Px}$) accruing to remaining lifetime production for survivors at age *x*, discounted by future mortality and scaled relative to the mean population growth rate (λ).

Approach 1 (simple) OUTPUT:

101 columns for different starvation curvature parameter γ ($0 < \gamma < 1$) and rows for each age (*x*) up to the maximum age (*xmax*) in the dataset:

Approach 2 (detailed) OUTPUT:

xmax rows (age *x*), columns concatenate three dimensions (γ = 0:01:1, r_x = 0:0.01:0.5, *k* = 0:0.1:1).

To obtain 2D tables (*x*, γ), data must be re-concatenated into 4D.
